# Epidermal PPARγ Signaling as a Suppressor of Toll-like Receptor-Mediated Inflammation and Fibrosis: Relevance to Cutaneous Squamous Cell Carcinoma

**DOI:** 10.3390/ijms27094136

**Published:** 2026-05-05

**Authors:** Raymond L. Konger, Ethel Derr-Yellin

**Affiliations:** 1 Department of Pathology & Laboratory Medicine, Indiana University School of Medicine, Indianapolis, IN 46202, USA; ederryel@iu.edu; 2 Department of Dermatology, Indiana University School of Medicine, Indianapolis, IN 46202, USA; 3 Richard L. Roudebush Veterans Affairs Hospital, Indianapolis, IN 46202, USA

**Keywords:** peroxisome proliferators activated receptor gamma, toll-like receptors, inflammation, tumor–stromal crosstalk, tumor microenvironment, non-melanoma skin cancer, fibrosis

## Abstract

Mice lacking epidermal *Pparg* (*Pparg*-/-^epi^) exhibit increased cutaneous carcinogenesis, while PPARγ signaling is reduced in actinic keratoses (AKs) and cutaneous squamous cell carcinomas (cSCCs). Using transcriptomic analysis, we now show that the top upregulated genes in *Pparg*-/-^epi^ mouse skin, human AKs and cSCCs encode multiple damage-associated molecular patterns (DAMPs) that are TLR4 ligands, while the TLR4 agonist lipopolysaccharide (LPS) is also predicted to be the top common activated upstream regulator in both *Pparg*-/-^epi^ mouse skin and in tumor datasets. By single-cell sequencing, DAMP expression was particularly elevated in myeloid cells and myofibroblasts of *Pparg*-/-^epi^ mice, and these cell types exhibit transcriptional changes consistent with TLR4 signaling. Myeloid cells also exhibited a loss of *Pparg* expression and activity. Transcriptional analysis of published LPS-treated macrophages also reveals a decrease in PPARγ activity. Fibroblasts from *Pparg*-/-^epi^ mice included cells with a gene expression profile resembling myofibroblasts found in cancer and fibrotic diseases. This was accompanied by increased dermal fibrosis in aged mice and a transcriptomic profile that indicates a key role for both TLR4 and TGFβ signaling. These data suggest that loss of epidermal PPARγ may disrupt counterbalancing PPARγ–TLR4 signals, leading to chronic inflammation and fibrosis, hallmarks of cutaneous neoplasia.

## 1. Introduction

Peroxisome proliferator-activated receptors (PPARs) are members of a large family of related ligand-activated nuclear transcription factors (for a review, see [1]). Three different PPAR proteins have been identified (PPARα, PPARδ and PPARγ). PPARs bind to DNA target sequences as heterodimers with the retinoid X receptor α (RXRα). PPARγ plays a key role in glucose homeostasis and adipogenesis [2,3]. This has led to the use of PPARγ agonists (e.g., rosiglitazone and pioglitazone) as anti-diabetic agents. PPARγ activation also suppresses the activity of transcription factors such as nuclear factor kappa-light-chain-enhancer of activated B-cells (NF-κB), activator protein 1 (AP-1), and nuclear factor of activated T cells (NFAT) [4,5]. In addition, loss of PPARγ expression is associated with human inflammatory skin disease [6,7].

In mice, keratinocyte-specific loss of *Pparg* (*Pparg*-/-^epi^ mice) results in increased cutaneous carcinogenesis and photoinflammation [8], a severe defect in normal contact hypersensitivity (CHS) responses [9], and the development of spontaneous inflammatory skin lesions [10]. Rosiglitazone suppresses chemical carcinogenesis [3], blocks the ability of ultraviolet light to suppress both CHS responses and anti-tumor immunity [9], and promotes anti-tumor immune reactions [11]. Transcriptomic analysis of differentially expressed genes in *Pparg*-/-^epi^ mice relative to wildtype control mice indicates that epidermal PPARγ acts as an important anti-inflammatory signal [10].

Cancer develops within an inflammatory microenvironment [12,13]. Persistent activation of NF-κB and Pattern Recognition Receptors (PRRs) are characteristic features of the cells within both chronic inflammatory lesions and tumor stroma [14,15]. Toll-like receptors (TLRs) are PRRs that play key roles in inflammation [15]. Of the TLRs, TLR4 has drawn particular interest as a potential target for skin cancer prevention and treatment [16]. In mice, TLR4 expression is directly correlated with UV-induced tumor yield, inflammatory mediator expression and CD11b^+^Gr-1^+^ myeloid cell recruitment [17]. Activation of PRRs occurs through the binding of a number of mediators produced by pathogenic microorganisms (pathogen-associated molecular patterns (PAMPs)) or as a result of cell damage (damage-associated molecular patterns (DAMPs)) [15].

TLR activation leads to canonical activation of NF-κB [15,16]. NF-κB activation in turn triggers the transcription of numerous cytokines/chemokines, including interleukin (IL)-1β and tumor necrosis factor (TNF)-α, as well as genes encoding DAMPs. These inflammatory mediators act to further induce NF-κB activation, both in the cell of origin or other cells [15,16]. Thus, unopposed TLR4 activation can trigger a feed-forward mechanism that maintains a chronic inflammatory state. In skin, chronic TLR4 activation results not only in inflammation but also fibrosis and a transforming growth factor (TGF)β-dependent gene signature [18].

PPAR signaling has been shown to be the top common inhibited canonical signaling pathway in mouse and human cutaneous squamous cell carcinomas (SCCs) and human actinic keratoses (AKs) [19]. Loss of PPAR signaling was accompanied by a reduction in *PPARG* expression and, to a lesser extent, *PPARA* transcript expression in both whole tumors as well as microdissected tumor epithelium [19]. Gene set enrichment analysis also predicted a reduction in PPARγ signaling for both AKs and SCCs [19]. The changes in PPAR signaling and PPARγ mRNA expression and activity were tumor-specific, as they are not observed in sun-exposed skin (SES) relative to non-exposed skin (NES) [19].

The above evidence suggests that the loss of PPARγ activity in cutaneous malignancy may be tied to the chronic inflammatory microenvironment that is observed in malignancy. We therefore sought to understand how loss of PPARγ activity within the epithelium triggers inflammatory changes in the dermis. Using gene set enrichment analysis of whole transcriptomic and single-cell RNA sequencing, we now show that there is considerable overlap in upstream signaling molecules that are activated in non-melanoma skin cancer (NMSC) and *Pparg*-/-^epi^ mice. Our data suggest that loss of *PPARG* expression in NMSC results in unopposed inflammatory signaling that provides a supportive environment for tumor development. Our findings also suggest a key role for DAMPs, TLR4 and TGFβ as important downstream drivers for the observed changes in both *Pparg*-/-^epi^ mouse skin as well as NMSC.

## 2. Results and Discussion

### 2.1. Pparg-/-^epi^ Mice Share Top Upstream Regulators with AKs and SCCs but Not with Sun-Exposed Skin: The Top Common Upstream Regulator Is LPS

Using gene set enrichment analysis of whole transcriptomic data, the top common inhibited canonical pathway in AK and SCC datasets is PPAR signaling [19]. Human AKs, human SCCs, and mouse SCCs also exhibited reductions in PPARγ mRNA expression of 39.60%, 62.29% and 84.66%, respectively [19]. In contrast, in human sun-exposed skin (SES), relative to non-exposed skin (NES), *PPARG* expression was increased by 50.18% [19]. Finally, transcriptomic analysis showed that PPARγ is predicted to be inhibited in both human and mouse SCC datasets but not SES [19].

**Figure 1:**
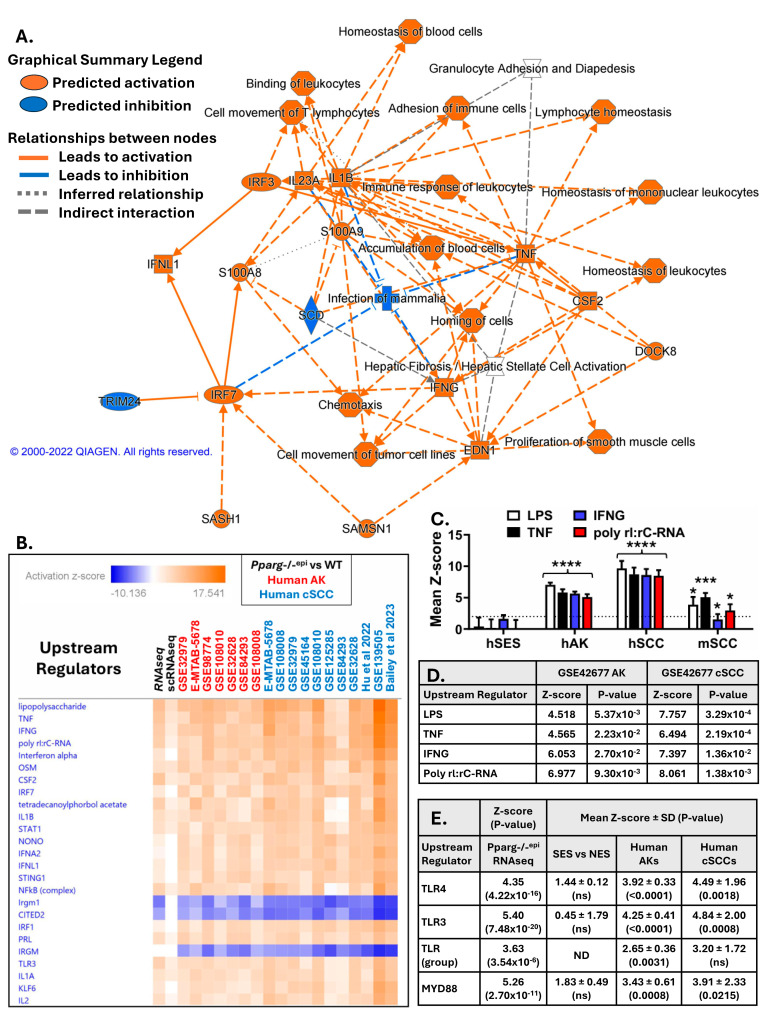
**LPS signaling is the top activated upstream regulator in both *Pparg*-/-^epi^ mouse skin and human actinic keratoses (AKs) and cutaneous SCC (cSCC).** (**A**) Differentially expressed genes from whole transcriptomic mRNA datasets of wildtype and *Pparg*-/-^epi^ mouse skin were uploaded for Ingenuity Pathway Analysis (Qiagen). The graphic details the positive and negative interactions between Canonical Signaling Pathways, Upstream Regulators, and Diseases and Functions. Critical nodal influences are shown along with the predicted activation state of each node as well as the role each node plays in activating or inhibiting other critical nodal influences. (**B**) Qiagen Ingenuity Pathway Analysis was utilized to perform an analysis of potential upstream regulatory elements that are either activated (brown tones) or inhibited (blue tones) in the different datasets. (**C**) For each dataset, the activation z-score was obtained for the top 4 upstream regulators (lipopolysaccharide (LPS), tumor necrosis factor alpha (TNF), interferon gamma (IFNG) and the TLR3 synthetic agonist, poly rI:rC-RNA. The data shown are the mean and SEM for the different z-scores for each of the control sun-exposed skin (relative to non-exposed), and human AK (hAK), human SCC (hSCC) and mouse SCC (mSCC). The hashed line represents the cutoff for predicting activation of an upstream regulator (z-score of 2.0). *, *p* < 0.05; ***, *p* <0.001; ****, *p* < 0.0001, values significantly different from 0, 1 sample *t*-test. A 1-sample *t*-test analysis to assess whether the mean z-scores were significantly different from the z-score cutoff of 2.0 was also performed: For LPS, *p*-values were <0.0001 for both hAK and hSCC. For TNF, *p*-values were 0.0003, 0.0001, and 0.0023 for hAK, hSCC and mSCC, respectively. For IFNG, *p*-values were <0.0001 for both hAK and hSCC. For poly rI:rC-RNA, *p*-values were 0.0004 and <0.0001 for hAK and hSCC. (**D**) Transcriptomic data obtained from microdissected tumor epithelium from actinic keratoses (AKs) and cutaneous SCC (cSCC) were downloaded from the gene expression omnibus [20]. An analysis of this publicly available dataset predicts that LPS, TNF, IFNG and poly rI:rC-RNA upstream regulators are activated (z-score > 2.0, *p*-value < 0.05). (**E**) Toll-like receptor (TLR) activation is seen in both *Pparg*-/-^epi^ mouse skin and human AKs and SCCs but not SES. A gene set enrichment analysis for upstream regulator influences was performed for the whole transcriptomic *Pparg*-/-^epi^ dataset as well as the 7 AK datasets and 11 SCC datasets shown in Figure 1B. We also analyzed the four SES relative to NES datasets. The activation z-score and *p*-value for the *Pparg*-/-^epi^ dataset is shown. For the SES and tumor datasets, the z-score is shown as the mean and SD. *p*-values for the tumor and SES datasets were performed using a 1-tailed *t*-test, analyzing whether the mean z-scores were significantly different from 2.0 (the cutoff for predicting activation of the upstream regulator).

If loss of PPAR signaling, and particularly PPARγ signaling, is a key feature of cutaneous malignancy, it is still unclear what role PPARγ is playing in tumor biology. However, clues to this role are provided by a more in-depth analysis of the differentially expressed genes found in *Pparg*-/-^epi^ mouse skin. Figure 1A depicts how loss of epidermal *Pparg* alters inflammatory cell infiltration and fibrosis. The calgranulins (S100A8 and S100A9), interleukin (IL)1β, tumor necrosis factor (TNF)α, interferon (IFN)γ, IL23A, colony-stimulating factor 2 (CSF2), and endothelin 1 (EDN1) all represent upstream regulators with prominent hub influences. Thus, while the loss of PPARγ expression is restricted to the epidermis, the greatest effect was associated with changes in the stromal microenvironment.

We next analyzed the *Pparg*-/-^epi^ mouse skin and human AK and SCC transcriptomic datasets to identify common activated or inhibited upstream regulators. This analysis showed a high degree of similarity (Figure 1B). The top four activated upstream regulators were the TLR4 ligand lipopolysaccharide (LPS), TNF, interferon gamma (IFNG), and the TLR3 ligand poly rI:rC-RNA. In Figure 1C, we plotted the mean activation z-scores for these top four upstream regulators in tumors and sun-exposed skin. There was a tumor-specific increase in the activation scores for all four regulators in human actinic disease and SCC datasets, but not sun-exposed skin (SES). The data were not as strong in mouse tumors, as only the mean z-score for TNF was significantly greater than 2.0. We also found that all four of these upstream regulators are predicted to be activated after reanalysis of a publicly available microdissected tumor epithelium dataset (Figure 1D) (GSE42677 [20]). These data support a key role for TLRs, particularly TLR4, in mediating both tumor biology as well as changes in the skin of *Pparg*-/-^epi^ mice.

As LPS is a TLR4 ligand, we further examined whether upstream regulator analysis would predict activation of the TLR system in *Pparg*-/-^epi^ mouse skin as well as the tumor datasets (Figure 1E). The analysis predicted that TLRs as a group, and TLR4 and TLR3 individually, were activated, with z-scores significantly greater than the activation cutoff of 2.0. MYD88 acts downstream of TLRs to stimulate TRAF6-dependent inflammatory mediator and type I interferon production [21]. We therefore show that MyD88 is predicted to be activated in *Pparg*-/-^epi^ mouse skin as well as the tumor datasets. As noted above, sun-exposed skin does not demonstrate a decrease in PPARγ expression or activity and lacks a predicted increase in LPS signaling (Figure 1C). Consistent with these data, the analysis of the SES datasets also failed to show a significant activation of TLRs or MyD88 (Figure 1E).

Additional upstream regulators that are predicted to be activated in Figure 1B include NF-κB (complex), IL-1β and interferon regulatory factor 7 (IRF7). An increase in NF-κB complex signaling and IL-1β production would be expected with the activation of receptors for LPS, the TLR3 agonist poly rI:rC-RNA, and TNFα [22,23,24]. Similarly, activated IRF7 drives the expression of type I interferons downstream of TLR activation [25,26].

In Figure 1B, gene set enrichment analysis also identifies two upstream regulators predicted to be strongly inhibited in both *Pparg*-/-^epi^ mouse skin and human AKs and SCCs. Immunity-related GTPase family M protein (*IRGM*/Irgm1) plays a key role in regulating mitochondrial autophagy [27]. Downregulation or loss of IRGM1 results in the release of mitochondrial DAMPs (e.g., mitochondrial DNA) that activate cellular pattern-recognition receptors, leading to the release of inflammatory cytokines [27]. Cbp/P300-interacting transactivator with Glu/Asp-rich carboxy-terminal domain 2 (CITED2) is a transcription factor cofactor that can interact with PPARγ as well as other transcription factors [28]. CITED2 appears to play an important role in cellular proliferation, stem cell renewal and cancer metastasis [28]. In macrophages, CITED2 acts as a general suppressor of inflammatory signaling pathways [29,30]. Moreover, loss of CITED2 results in upregulated inflammatory signaling pathways, while overexpression blocks LPS-induced NF-kB activation [29]. The mechanism by which loss of epidermal *Pparg* results in reduced activity of these pathways remains unclear. Nonetheless, this reduced activity is consistent with the observed increase in inflammatory signaling.

### 2.2. Damage-Associated Molecular Patterns (DAMPs) with TLR4 Ligand Activity Are Among the Top Upregulated Genes in Pparg-/-^epi^ Mice, Actinic Disease and Mouse and Human cSCC

**Figure 2:**
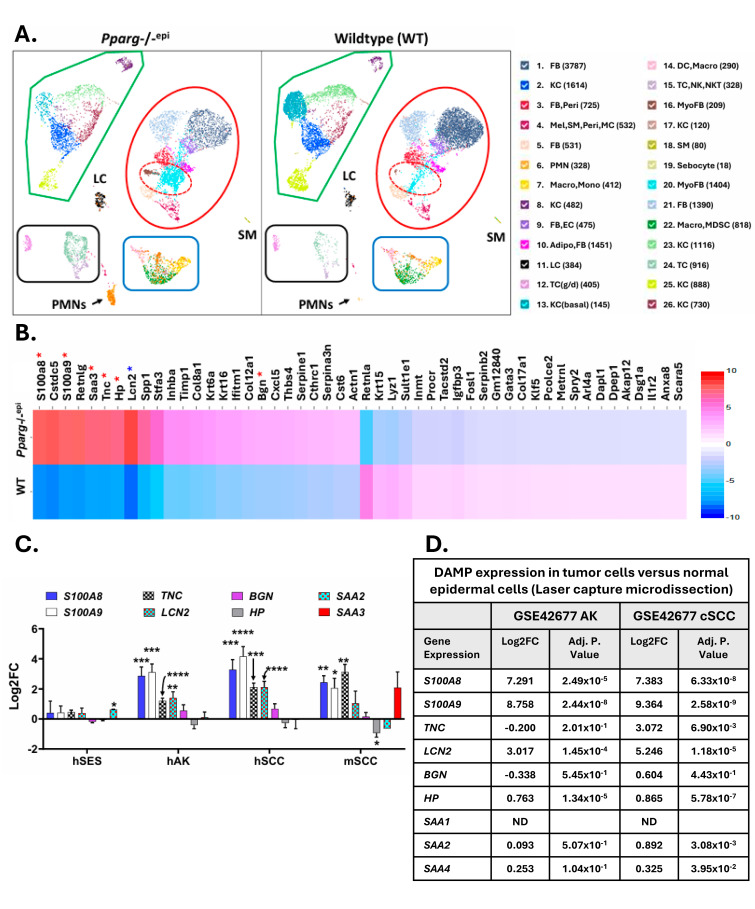
**Damage-associated molecular patterns are top upregulated genes in single-cell data from *Pparg*-/-^epi^ mice and are also a feature of NMSC.** (**A**) Uniform manifold approximation and projection (UMAP) plot of cells isolated from *Pparg*-/-^epi^ mice (left panel) or wildtype mice (right panel). *Pparg*-/-^epi^ cells (10,228 cells, with 2989 median identified genes per cell) and WT cells (12,699 cells, with 2489 median genes per cell) are segmented into 26 identified cell clusters (cell identification by color coding key on the right). Keratinocyte clusters are bordered by the green outline, fibroblast and other stromal cell populations are bordered by the red oval, myofibroblast clusters 16 and 20 are marked by the hashed red oval, non-granulocytic myeloid cells are bordered by the blue rectangle, and lymphoid cells are bordered by the black rectangle. Individual cell clusters containing granulocytes (PMNs), Langerhans cells (LCs) and smooth muscle cells (SMs) are also identified. Other abbreviations: fibroblast (FB), keratinocyte (KC), pericyte (Peri), melanocyte (Mel), mast cell (MC), macrophage (Macro), monocyte (Mono), endothelial cell (EC), adipocyte (Adipo), γ/δ T-cell (TC (g/d)), dendritic cell (DC), T cell (TC), natural killer cell (NK), natural killer T cell (NKT), myofibroblast (MyoFB), myeloid derived suppressor cell (MDSC). (**B**) Heat map showing the top 25 differentially expressed genes that are upregulated or downregulated in *Pparg*-/-^epi^ relative to wildtype mouse cells (scRNAseq dataset that includes gene expression data from all cell populations). The color bar scale to the right shows gene expression differences as a Log2-fold change. Red asterisks indicate genes with known TLR4 ligand activity. The blue asterisk indicates a gene that indirectly activates TLR4. (**C**) Gene expression levels for the DAMPs seen in B were obtained from publicly available transcriptomic datasets of human sun-exposed skin (hSES), human actinic keratoses (hAKs), human cutaneous squamous cell carcinoma (hSCC) and mouse cutaneous SCC (mSCC). For S100A8/A9, SAA1 and SAA2, TNC, HP, LCN2 and BGN, the data were obtained from the following number of datasets: hSES, n = 3 datasets; human actinic keratoses (hAKs), n = 7–10 datasets; and human and mouse SCC: hSCC, n = 11–14 datasets, and mSCC, n = 7–8 datasets, respectively. For the mouse homolog of SAA1, SAA3 data were obtained from n = 8 mSCC datasets. Data are plotted as the mean and SEM. Significantly different from 0 (1-sample *t*-test): *, *p* < 0.05; **, *p* < 0.01; ***, *p* < 0.001; ****, *p* < 0.0001. (**D**) Expression of DAMP mRNAs in actinic keratoses (AKs) and human cutaneous SCCs (cSCCs) from a study that obtained tumor epithelium by microdissection [20]. Data are shown as a log 2-fold change (Log2FC) with adjusted *p*-values (Adj *p*. Value).

We recently performed single-cell RNA sequencing (scRNAseq) of wildtype and *Pparg*-/-^epi^ mouse skin [19]. In Figure 2A, uniform manifold approximation and projection (UMAP) plots are shown depicting the identified cell clusters that were found in both mouse genotypes. As reported previously [19], *Pparg*-/-^epi^ mice have increased numbers of both myeloid cells and lymphocytes. We have also demonstrated that fibroblasts expressing the myofibroblast marker genes *S100a4* and *Acta2* are enriched in *Pparg*-/-^epi^ mice relative to WT mice (31.29% and 6.96% of non-immune stromal cells respectively) [19]. In Figure 2A, these myofibroblast clusters (clusters 16 and 20) are outlined with a hashed red oval.

Following scRNAseq, Figure 2B depicts a heat map that highlights the top 25 differentially expressed transcripts in *Pparg*-/-^epi^ mouse cells relative to WT mice. Overall, six of the top 25 differentially expressed transcripts are known DAMPs with TLR4 ligand activity (red asterisks in Figure 2B): the calgranulins S100A8 and S100A9 [31], serum amyloid A3 (*Saa3*) [32], tenascin-C (*Tnc*) [33,34]*,* haptoglobin (*Hp*) [35], and biglycan (*Bgn*) [36].

In addition to these DAMPs with direct TLR4 ligand activity, lipocalin 2 (*Lcn2*) was also shown to be differentially upregulated in *Pparg*-/-^epi^ mice (blue asterisk in Figure 2B). While lipocalin 2 is not a TLR ligand, it activates TLR2 and TLR4 signaling indirectly by inducing the release of the TLR2 and 4 agonist high mobility group box 1 (HMGB1) [37,38]. Additional evidence for TLR4 activation in *Pparg*-/-^epi^ mice is seen by the upregulated expression of the LPS-inducible gene products stefin A3 (*Stfa3*) and osteopontin (*Spp1*) (Figure 2B) [18,39].

TLR4 activation is known to induce both *Saa3* [32] and *Lcn2* [40] expression, suggesting a potential feed-forward mechanism for chronic activation of TLR4. This type of feed-forward TLR4 signaling system has been seen with a calprotectin/SAA*3*/TLR4 pathway in mouse lung cancer [32]. It should be noted that the human homologue of the mouse *Saa3* gene is a pseudogene (*SAA3P*). However, the mouse SAA3 protein is related in structure and function with the human and mouse SAA1, SAA2 and SAA4 proteins [41]. All of the SAA isoforms are ligands for TLR4 [42].

In Figure 2C, we show the expression of these DAMPs in transcriptomic datasets of human SES, AKs and SCCs, as well as mouse SCCs. Modest or insignificant changes in DAMP transcripts were noted for SES relative to NES skin. In contrast, the calgranulins, *TNC* and *LCN2* were significantly elevated in both hAKs and hSCCs. In mouse SCCs, the calgranulins and *Tnc* were significantly increased, while *Lcn2* was not. For *TNC*, these transcriptomic data agree with a study that demonstrated a significant elevation in TNC protein in an AK and SCC relative to normal skin [43]. A modest but significant increase in *SAA2* was observed in human SES skin (Figure 2C).

The data in Figure 2C represent whole tumor transcriptomic data. To determine whether these DAMPs are increased in isolated tumor cells, we examined the expression of DAMP mRNA in microdissected tumor epithelium [20] (Figure 2D). These data showed increases in *S100A8*, *S100a9*, *LCN2* and *HP* in AKs and SCCs. In SCCs, *TNC* and *HP* expression were also increased.

It should be noted that these DAMPs can also activate other receptors. S100A8/A9 can activate the receptor for advanced glycation end products (RAGE/*AGER*) [44]. SAA3 and other SAA proteins are ligands for not only TLR4 but also TLR2 and the scavenger receptor CD36 [45]. However, CD36 can serve to promote ligand interaction with TLR2 and TLR4 and also promotes TLR activation [46] and elicits sterile inflammation by ligand-induced formation of TLR 4 and 6 heterodimers [47]. Biglycan, like SAA3, can activate both TLR2 and 4 [36]. TNC can activate both TLR4 and integrin α9β1 [33]. Finally, HP is also a low-affinity ligand for CD11b/CD18 [48]. Thus, a role for these other receptors in mediating the observed changes in *Pparg*-/-^epi^ mouse skin is also possible.

### 2.3. DAMP Expression Is Enriched in Myeloid and Fibroblast Clusters of Pparg-/-^epi^ Mice

**Figure 3:**
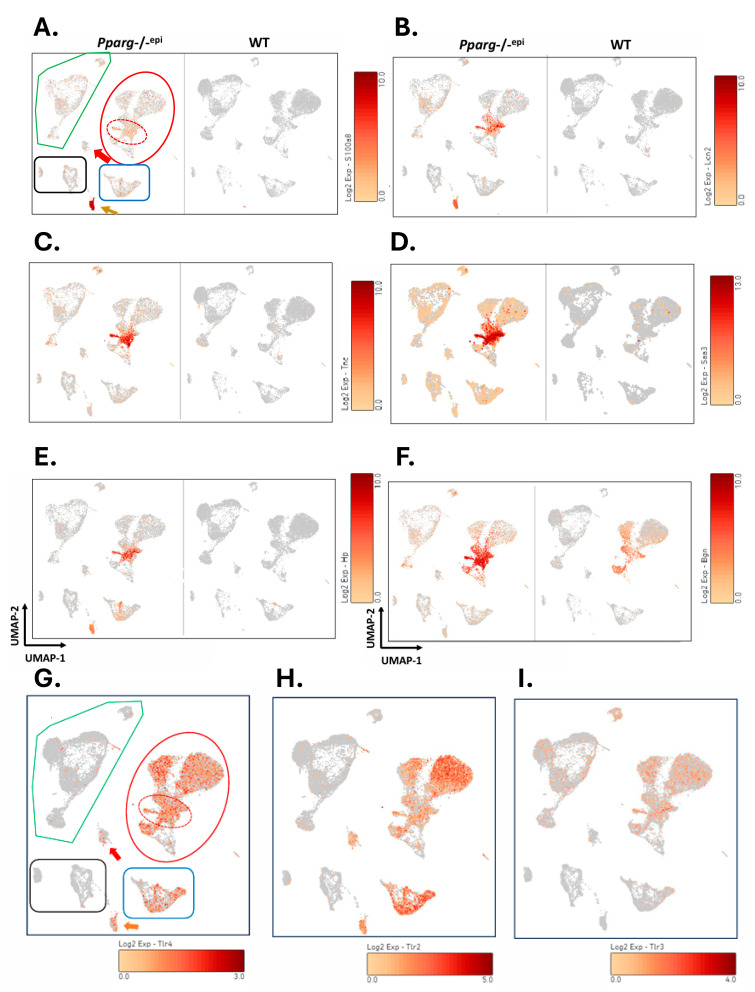
**Cluster-specific differences in DAMP expression in *Pparg*-/-^epi^ and wildtype (WT) mouse skin cells.** Expression data are shown in UMAP cell clusters by color mapping, with expression depicted using a color scale bar (Log2-fold change (Log2 Exp) for each transcript). (**A**) Expression of S100A8 in *Pparg*-/-^epi^ and WT cell clusters. In panels (**A**,**G**), the colored outlines are used to border specific cell clusters: keratinocyte clusters (green); non-immune cell, non-smooth muscle stromal cell clusters (solid red); myofibroblasts (hashed red outline); non-neutrophil myeloid cell clusters (except for Langerhans cells) (blue); *Cd3*+ cells (black). The brown arrow points out the neutrophil cluster, while the red arrow points to the Langerhans cells. (**B**–**F**). UMAPs depicting the expression level of *Lcn2* (**B**), *Tnc* (**C**), *Saa3* (**D**), *Hp* (**E**) and *Bgn* (**F**) in each cluster. (**G**–**I**). UMAPs depicting the expression level of *Tlr4* (**G**), *Tlr2* (**H**) and *Tlr3* (**I**) in the different cellular compartments.

In Figure 3A–F, we show cell type-specific expression data for these DAMPs in both WT and *Pparg*-/-^epi^ mouse skin. Non-immune stromal cell clusters from *Pparg*-/-^epi^ mouse skin were enriched in *S100a8* (Figure 3A), *Lcn2* (Figure 3B), *Tnc* (Figure 3C), *Saa3* (Figure 3D), *Hp* (Figure 3E) and *Bgn* (Figure 3F). *S100a9* expression essentially mimicked the expression pattern of *S100a8* (not shown). The highest level of expression of these DAMPs co-localized to FB clusters expressing myofibroblast markers (identified by the hashed red oval in Figure 3A). Neutrophils from *Pparg*-/-^epi^ mice also expressed higher levels of *S100a8*, *Lcn2*, *Saa3* and *Hp*. Interestingly, both myofibroblasts and neutrophils are two of the cell populations that are increased the most dramatically in *Pparg*-/-^epi^ mouse skin, with a 43-fold increase in neutrophils and a 4.5-fold increase in fibroblasts that express myofibroblast marker genes [19].

As these DAMPs are known to activate TLR4, and in some cases TLR2, we examined the expression of these two receptors in the different cell clusters. Both *Tlr4* (Figure 3G) and *Tlr2* (Figure 3H) expressions are increased in myeloid and fibroblast cell clusters relative to keratinocytes and T-cell clusters. Thus, increased DAMP expression should be of particular importance to myeloid and fibroblast cells. This is interesting in that chronic TLR2 and 4 activation in fibroblasts and macrophages are associated with the conversion to cancer-associated fibroblast (CAF) and cancer-associated macrophage populations [23].

In Figure 1B,C, the TLR3 agonist poly rI:rC-RNA was predicted to be a top activated upstream regulator in both *Pparg*-/-^epi^ mouse skin and the tumor datasets. In Figure 3I, we show that TLR3 expression is greatest in non-immune stromal cell populations and, to a lesser extent, keratinocytes. The expression of TLR3 in keratinocytes is interesting as TLR3 activity has been shown to be important for barrier function recovery following UV treatments [49].

LPS recognition by TLR4 occurs through receptor complexes that includes myeloid differentiation-2 (MD-2, *Ly96*), CD14 or CD36 [46]. Significantly less is known about how endogenous TLR ligands interact with the receptor. However, SAA3 can bind directly to MD-2 to trigger TLR4-dependent MyD88 activation [50]. As noted above, SAA3 is also a ligand for CD36 as well as TLRs 2 and 4. We therefore examined the expression pattern of the TLR4 co-receptor molecules *Cd14* (Figure S1A) and *Ly96* (Figure S1B) and *Cd36* (Figure S1C). Macrophage/monocytes heavily expressed all three co-receptor transcripts, while neutrophils expressed high levels of *Cd14*, lower levels of *Ly96* and little *Cd36*. Fibroblasts expressed relatively high levels of *Ly96*, modest levels of *Cd14*, and largely absent *Cd36* expression. Thus, if protein levels correspond to transcript levels, it would be expected that myeloid populations should have more robust TLR4-dependent signaling in response to DAMPs.

Finally, we examined the cell clusters that are associated with the other top upregulated genes that were seen to differentiate *Pparg*-/-^epi^ mouse cells from wildtype mouse skin cells (Figure 2B). Of interest, the myofibroblast clusters seen in Figure 2B (clusters 16 and 20) had high levels of expression of many of these genes. The following were enriched in clusters 16 and 20 (Figure S2A–K): *Spp1*, *Inhba*, *Timp1*, *Col8a1*, *Col12a1*, *Cxcl5*, *Thbs4*, *Serpine1*, *Cthrc1*, *Serpina3n* and *Actn1*. This suggests that these fibroblast clusters play an important role in the skin phenotype of *Pparg*-/-^epi^ mice. The remaining top upregulated genes in Figure 2B for *Pparg*-/-^epi^ mice were found primarily in either keratinocyte clusters (*Cstdc5*, *Stfa3*, *Krt6a*, *Krt16*, and *Cst6*) or the neutrophil cluster (*Retnlg* and *Ifitm1*) (not shown).

### 2.4. Pparg-/-^epi^ Mouse Dermal Macrophages and Langerin-Positive and -Negative Dendritic Cell Populations Show Distinct Differences in Transcriptomic Profiles

Myeloid cell populations exhibit significant heterogeneity. We therefore performed a recluster analysis of the neutrophils (cluster 6), Langerhans cells (cluster 11), and macrophages/dendritic cells (clusters 7, 14 and 22) that are seen in Figure 2A. In the UMAP plot of the reclustered myeloid cells shown in Figure S3A, WT and *Pparg*-/-^epi^ mouse skin macrophages, dendritic cells and the Langerhans cells had largely distinct transcriptomic profiles. As neutrophils were nearly absent in the WT dermis, it was not possible to determine differences between WT and *Pparg*-/-^epi^ neutrophils.

In Figure S3B, a heat map shows the top genes that differentiate the reclustered WT and *Pparg*-/-^epi^ myeloid cells. The DAMPs *S100A8*, *S100A9*, *Lcn2*, and *Hp* were among the top upregulated genes in the *Pparg*-/-^epi^ myeloid cell populations. However, as neutrophils heavily express all four of these DAMPs (Figure 3), the nearly exclusive presence of neutrophils in *Pparg*-/-^epi^ mouse skin would be expected to skew the results. To remove the influence of the neutrophils from the analysis, we performed subcluster analyses of the macrophage/dendritic cell populations alone (clusters 7, 14 and 22 from Figure 2A). A separate subcluster analysis of Langerhans cells was also performed. Due to the lack of control WT neutrophils, a comparative analysis of *Pparg*-/-^epi^ relative to WT neutrophils was not possible.

Following subclustering of the macrophage/DC clusters 7, 14 and 22 from Figure 2A, we again show distinct segregation of the WT and *Pparg*-/-^epi^ macrophages and DCs (Figure S4A). Absent contributions from neutrophils, the top 5 upregulated transcripts in *Pparg*-/-^epi^ macrophage/DC cells relative to WT cells still included the calgranulins (*S100a8*/*a9*). The *Pparg*-/-^epi^ macrophage/DC cells were also differentiated by increased expression of osteopontin (*Spp1*), chitinase-like 3 (*Chil3*) and secretory leukocyte protease inhibitor (*Slpi*) (Figure S4B). These genes are known to be increased in monocyte-derived macrophages that are recruited to sites of inflammation [51,52].

In contrast, wildtype myeloid clusters expressed high levels of resistin-like alpha (*Retnla*); lysozyme 1 (*Lyz1*); eosinophil-associated, ribonuclease A family, member 2 (*Ear2*); and a mouse homologue of the human DC-specific ICAM-3-grabbing non-integrin (DC-SIGN; CD209) gene (*Cd209f*) (Figure S4B). The first three of these markers have also been associated with monocyte-derived macrophages [53]. Lysozyme 1 is also associated with dendritic cell maturation [51]*. Cd209f* was also found to be expressed in two different macrophage clusters in normal mouse dermis [54].

In Figure S4C, reclustering revealed six different cell subcluster populations. After Enrichr annotation, all clusters were identified as containing macrophages. However, scoring also matched with myeloid-derived suppressor cells (clusters 1 and 4), monocytes (clusters 2 and 6), and dendritic cells (clusters 2 and 3).

To further verify that clusters 2 and 3 contain high numbers of dendritic cells, we show that macrophage markers F4/80 (*Fcgr1*) (Figure S4D) and *Msr1* (not shown) are poorly expressed by clusters 2 and 3. Moreover, these two clusters also expressed genes that are associated with dendritic cell maturation and function. This included the expression of *Irf4* [55] in clusters 2, 3 and 5 (Figure S4E); basic leucine zipper ATF-like transcription factor 3 (*Batf*) [55] in cluster 2 (Figure S4F); and FMS-like tyrosine kinase 3 (*Flt3*) [56] in cluster 3 (Figure S4G). Although cluster 3 also had a weaker match with plasmacytoid DCs (pDCs), none of the clusters expressed the pDC marker *Ifna1* (not shown). This is also consistent with a previous study showing that mouse skin does not normally contain plasmacytoid DCs [57].

**Figure 4:**
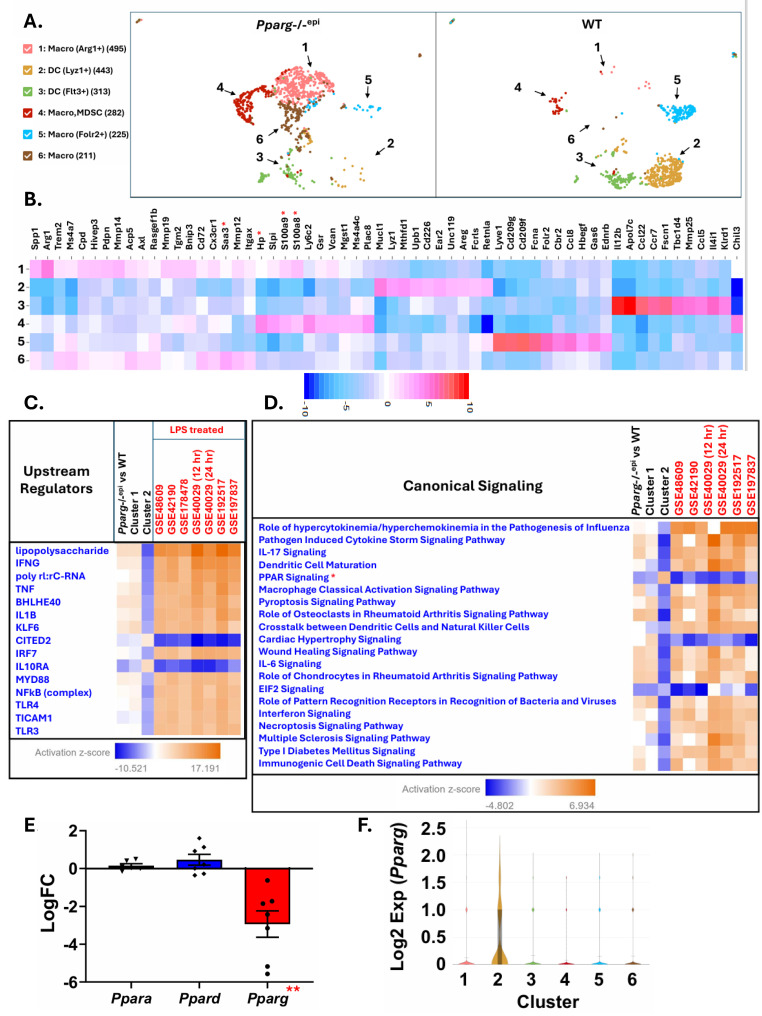
**Recluster analysis of non-granulocytic myeloid cell clusters from WT and *Pparg*-/-^epi^ mouse dermis.** Non-granulocytic myeloid cell clusters 7, 14 and 22 from Figure 1A were reclustered. (**A**). UMAP cluster depiction of *Pparg*-/-^epi^ myeloid populations (left panel) and wildtype (WT) myeloid cells (right panel). Reclustering resulted in 6 new clusters that were unequally distributed between WT and *Pparg*-/-^epi^ mouse skin. Abbreviations: Macro (macrophage); DC (dendritic cell); MDSC (myeloid-derived suppressor cell); *Arg1* (Arginase 1); *Flt3* (FMS-like tyrosine kinase 3); *Folr2* (folate receptor beta). (**B**). Heat map showing the top differentially expressed gene features that characterize the 6 different myeloid cell clusters from each other. DAMPs are marked by red asterisks. (**C**,**D**). Gene set enrichment analysis was performed using datasets of the differentially expressed genes for all macrophage/dendritic cells in *Pparg*-/-^epi^ relative to WT cells, differentially expressed genes specific to cluster 1 myeloid cells (largest cluster found predominantly in *Pparg*-/-^epi^ mouse skin), cluster 2 myeloid cells (largest cluster found primarily in WT mouse skin), and 6 publicly available transcriptomic datasets that examined the genes that are differentially expressed following LPS-treatment of macrophages. (**C**) Heat map showing the top 15 upstream regulators that were observed in all datasets (sorted by activation z-score). (**D**) Heat map showing the top 20 canonical signaling pathways for all datasets (sorted by activation z-score). PPAR signaling is the top common inhibited canonical signaling pathway in all datasets except for cluster 2 myeloid cells (noted by the red asterisk). (**E**). PPAR isotype mRNA expression was obtained for the LPS-treated macrophage sequencing studies. The mean change in PPAR expression for LPS-treated vs. control macrophages is shown (±SEM) (LogFC = Log2-fold change). **, *p* < 0.01; one-sample *t*-test. (**F**). Violin plot showing *Pparg* expression within each of the 6 macrophage/dermal DC cluster.

In Figure 4A, we show how the six different macrophage/DC cell clusters segregate between *Pparg*-/-^epi^ and WT mouse dermis. Cells found in clusters 1, 4 and 6 were primarily found in *Pparg*-/-^epi^ dermis, while clusters 2 and 5 were largely found in WT mouse skin. In Figure 4B, a heat map depicts the top 10 transcriptional features that differentiate the 6 different clusters. Overexpression of *S100a8* and *S100a9* was limited to *Pparg*-/-^epi^ mouse-specific clusters 1 and 4. Both calgranulins are normally expressed in neutrophils, monocytes and some dendritic cells but are lost during macrophage differentiation [58]. However, subsets of macrophages expressing the calgranulins have been observed in inflammatory lesions [59]. Within the tumor microenvironment, upregulation of the calgranulins is associated with monocytic myeloid-derived suppressor cell (MDSC) and M2 macrophage differentiation [58,60]. This is consistent with both clusters 1 and 4 exhibiting transcriptional features that annotate to MDSCs (Figure S4C).

Figure 4B also shows that the myeloid clusters that are found primarily in *Pparg*-/-^epi^ skin also expressed other DAMPs that are associated with TLR2/4 activity. *Saa3* was found to be expressed at high levels in cluster 6, while *Hp* was found to be highly expressed in cluster 4 cells. Of interest, *Saa3* expression is known to be induced in macrophages by LPS [61]. Similarly, clusters 1 and 4 differentially expressed high levels of *Slpi*, which is also known to be induced by LPS in macrophages [62].

Since macrophages in *Pparg*-/-^epi^ mice express TLR4 ligands and gene products that are known to be upregulated in LPS-treated macrophages, we performed gene set enrichment analysis of the differentially expressed genes found in *Pparg*-/-^epi^ dermal macrophages/dendritic cells (Macro/DCs) relative to WT mice. The top four upstream regulators that were predicted to be activated were LPS, IFNG, TNF, and IL-1β (z-scores of 4.057, 2.095, 1.984, and 2.038, respectively (not shown)). *p*-values of overlap for all four upstream regulators were highly significant (3.99 × 10^−73^, 2.54 × 10^−50^, 3.48 × 10^−39^, and 6.08 × 10^−39^, respectively).

We therefore examined how closely the differentially expressed genes (DEGs) in *Pparg*-/-^epi^ mouse Macro/DC cells mimic DEGs from 7 published transcriptomic studies of LPS-treated macrophages relative to control macrophages (Figure 4C). We also included in the analysis the DEGs that were found in the largest macrophage clusters that are found in either *Pparg*-/-^epi^ mice (cluster 1) or WT mice (cluster 2). There was a high degree of concordance for upstream regulators in the *Pparg*-/-^epi^ global Macro/DC cluster dataset, as well as the cluster 1 dataset, relative to the LPS-treated macrophages. As expected, LPS was predicted to be the top common activated upstream regulator not only for both the LPS-treated macrophage datasets but also the *Pparg*-/-^epi^ Macro/DCs and the cluster 1 macrophages (Figure 4C).

In Figure 4C, other top common upstream regulators that were predicted to be activated included IFNG, TNF, basic helix–loop–helix family member E40 (BHLHE40), IL-1β, Kruppel-like transcription factor 6 (KLF6) and MYD88. The increase in BHLHE40 activation is interesting as this transcription factor mediates LPS-induced pro-inflammatory and glycolytic gene expression in macrophages [63]. Similarly, in human and mouse macrophages the transcription factor KLF6 acts cooperatively with NF-kB to promote LPS-induced pro-inflammatory gene expression [64]. As NF-kB activation by TLRs requires MyD88 activation [65], it is notable that MyD88 and NF-kB (complex) signaling are predicted to be activated upstream regulators in both *Pparg*-/-^epi^ macrophages and LPS-treated macrophages (Figure 4C). Overall, the data indicate that loss of epidermal *Pparg* is associated with myeloid cell changes that would be expected with TLR4 activation.

In Figure 4D, we next analyzed canonical pathway signaling for the differentially expressed genes from the pooled dataset (*Pparg*-/-^epi^ relative to WT mice), as well as the largest clusters found in *Pparg*-/-^epi^ mice (cluster 1) or WT mice (cluster 2). We compared these datasets with the 7 transcriptomic studies that examined LPS treatment of macrophages. Again, there was good correlation between the canonical pathways for the *Pparg*-/-^epi^ myeloid cells overall as well as cluster 1 cells with the LPS-treated macrophage datasets.

In Figure 4D, the top canonical pathway that was predicted to be inhibited was PPAR signaling. In Figure 4E, we therefore plotted the mean expression levels of each PPAR isotype that were obtained from the transcriptomic datasets for LPS-treated macrophages. Only *PPARG* was found to be significantly reduced, with a mean reduction of approximately 87% (mean Log2FC = −2.932). This is consistent with data reported for mouse peritoneal macrophages and the mouse RAW 264.7 macrophage cell lines in vitro [66]. In these RAW 264.7 cells, stimulation with TLR2 or TLR4 ligands suppresses *Pparg* transcript expression by approximately 90% [66]. This repression required NF-kB activation and could not be reversed by subsequent PPARγ ligand treatment [66]. As noted above, KLF6 is predicted to be activated in both *Pparg*-/-^epi^ macrophages and LPS-treated macrophages (Figure 4C). This is interesting as a recent study demonstrated that the ability of LPS to suppress *PPARG* expression in macrophages requires KLF6 [64]. Given this discussion, it is not surprising that only cluster 2 cells found in WT mice express *Pparg* (Figure 4F).

Thus, given the high level of TLR4 activation that is predicted in established AKs and SCCs, PPARγ agonists may be ineffective therapeutic agents. However, as increased LPS activity and loss of PPARγ expression are not seen in non-cancerous sun-exposed skin, this may provide an answer as to why rosiglitazone has skin cancer chemopreventive activity [3]. It would be interesting to determine whether the combination of TLR4 antagonists with PPARγ agonists may provide cooperative activity as chemopreventive and chemotherapeutic agents in NMSC.

### 2.5. Langerhans Cells (LCs) from Pparg-/-^epi^ Mice Also Exhibit Transcriptomic Features Consistent with TLR Activation

**Figure 5:**
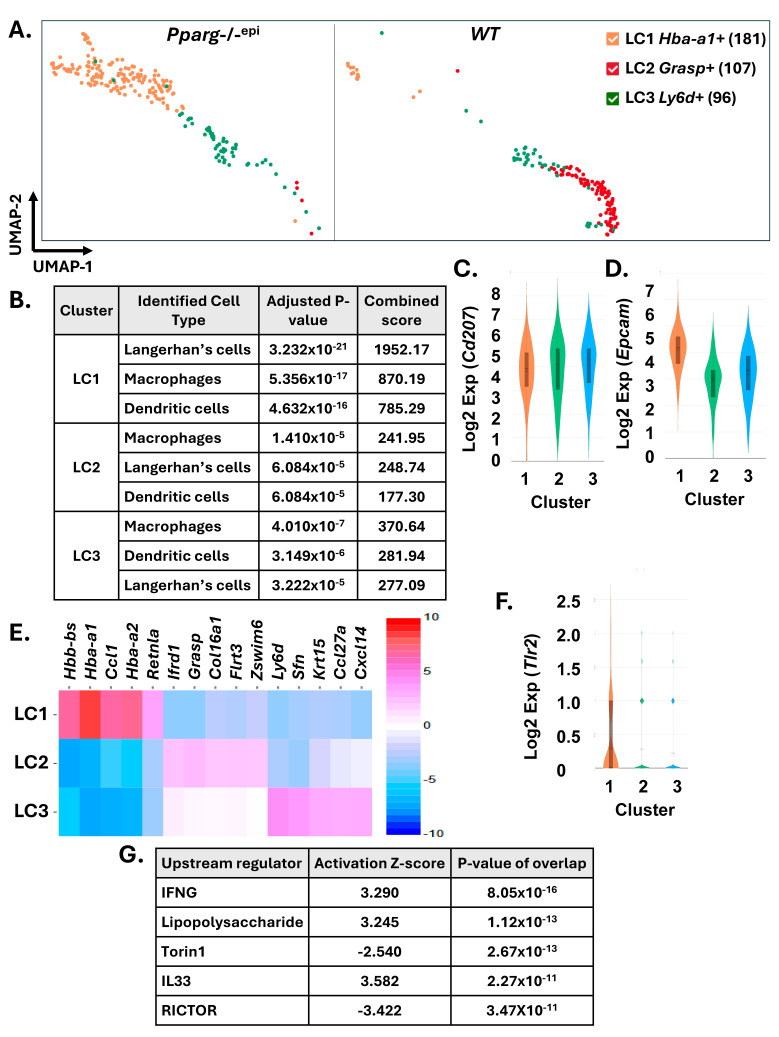
**Langerhans cell subclustering reveals three *Cd207*+ *Epcam*+ subclusters in which LPS and IFNG areas are again predicted to be activated in *Pparg*-/-^epi^ mouse skin.** (**A**) UMAP plot showing the unsupervised subcluster analysis of the Langerhans cell population from Figure 2A. Following reclustering, 3 clusters were identified. (**B**) The top differentiating gene features for all three clusters were analyzed by Enrichr for cell type analysis. (**C**,**D**) Violin plots indicate that all 3 subclusters expressed high levels of LC markers langerin (*Cd207*) (**C**) and *Epcam* (**D**). (**E**) Heat map showing the top 5 upregulated transcriptomic features that differentiate the three different LC subclusters (Log 2-fold change). (**F**) Expression of *Tlr2* in all 3 subclusters. (**G**) Following subclustering of *Cd207+* Langerhans cells, gene expression features that differentiated *Pparg*-/-^epi^ from WT LC cells were obtained and submitted for gene set enrichment analysis using Ingenuity Pathway Analysis (Qiagen). The table depicts the top 5 upstream regulators that are predicted to be activated or inhibited in *Pparg*-/-^epi^ LC subclusters relative to WT LCs. Ranked by *p*-value of overlap.

In Figure S3A, unsupervised cluster analysis showed clear separation of the LC cells found in WT and *Pparg*-/-^epi^ skin. After reclustering the LC cell cluster 11 from Figure 2A, three separate subclusters were revealed (Figure 5A). Of these three subclusters, cluster 1 cells were found primarily in *Pparg*-/-^epi^ mouse skin, while subcluster 2 cells were found largely in WT mouse skin.

In Figure 5B, we used Enrichr to identify the predominant cell types in each cluster. All three clusters were scored strongly for Langerhans cells as well as macrophages and dendritic cells. In Figure 5C, we verified that all three clusters expressed high levels of *Cd207* (Langerin), a marker of LCs and a subclass of dermal dendritic cells [67]. For cluster 3, the scoring was stronger for macrophages and dendritic cells, suggesting that some portion of this cluster contained langerin-positive dermal dendritic cells. However, all three clusters expressed high levels of *Epcam* (epithelial cellular adhesion molecule) (Figure 5D), a marker that distinguishes LCs from langerin+ dermal DCs [67].

In Figure 5E, top distinguishing transcriptomic features are shown for each of the three clusters. Interestingly, 3 of the 5 top upregulated genes in cluster 1 LCs were hemoglobin alpha and beta subunit genes (*Hbb-bs*, *Hba-a1*, and *Hba-a2*). Hemoglobin has been shown to be expressed in non-erythrocytic cells, including epithelial cells, macrophages, lymphocytes and dendritic cells [68]. In several types of cells, including human primary vaginal epithelial cells and macrophages, upregulation of hemoglobin can occur with LPS treatment or during sepsis [68].

Mouse LCs have been shown to express TLRs 2, 4 and 9 [69]. In Figure 3H, the *Tlr2* receptor was seen to be expressed in the LC cluster. In Figure 5F, we show that *Tlr2* expression was upregulated only in cluster 1 LC cells that are found in *Pparg*-/-^epi^ mouse skin. However, at the sensitivity of scRNAseq, none of the LC subclusters showed significant expression of *Tlr4* (data not shown). This absence of *Tlr4* expression in the LC cluster is also apparent in the image shown in Figure 3G.

After performing gene set enrichment analysis, LPS signaling is predicted to be one of the top activated upstream regulators in *Pparg*-/-^epi^ LC cells compared to their WT counterparts (Figure 5G). In Figure 3, *Saa3* expression was increased in LC cell populations in *Pparg*-/-^epi^ mice relative to WT mice. As SAA3 binds to TLR2, the increase in TLR2 expression in cluster 1 LC cells suggests the potential for autocrine activation of the LPS signaling pathway [70]. Additional paracrine signaling by DAMPs produced at high levels by other *Pparg*-/-^epi^ cells may also contribute to the activation of the LPS signaling pathway through TLR2 and possibly lower levels of TLR4.

In Figure 5G, interferon gamma (IFNG) and IL33 were also predicted to be top activated signaling pathways. IFNG treatment of human Langerhans cells promotes functional activation that is dependent on increased CD80/CD86 expression [71]. While no studies have examined the functional effects of IL33 signaling in Langerhans cells, IL33 is an alarmin that is associated with inflammatory skin diseases [72] and activates airway dendritic cells to stimulate a Th2 polarized lymphocyte response [73].

Figure 5G also shows that Rictor signaling and Torin1 activity were predicted to be inhibited in *Pparg*-/-^epi^ LCs. The rapamycin-insensitive companion of mammalian target of rapamycin (mTOR) (Rictor) is necessary for the formation of the active mammalian target of rapamycin complex 2 (mTORc2) [74]. Torin1 is a potent small molecule inhibitor of both mTORc1 and mTORc2 [75]. These data suggest that mTOR activity may also play a role in the observed changes to *Pparg*-/-^epi^ dermal LCs. Interestingly, while the role of Rictor in regulating LC function is not clear, loss of Rictor in dendritic cells promotes an increase in inflammatory mediator production following TLR4 activation [76].

### 2.6. Pparg-/-^epi^ Mouse Dermal Fibroblasts Exhibit a Profibrotic Signature That Is Transcriptionally Linked to TGFβ and TLR4 Signaling

In Figure S2, 10 of the top 25 gene transcripts that differentiate *Pparg*-/-^epi^ mice from WT mice were expressed in fibroblasts. It is notable that 9 of the 10 gene products (*Inhba* [77], *Timp1* [78], *Col8a1* [79], *Col12a1* [79], *Thbs4* [80], *Serpine1* [79], *Cthrc1* [77], *Serpina3n* [81] and *Actn1* [82]) are also induced by TGFβ or are TGFβ-induced cancer-associated fibroblast (CAF) markers. The tenth fibroblast gene transcript, osteopontin (*Spp1*), is necessary for TGFβ-induced fibrosis and fibroblast activation [83,84].

**Figure 6:**
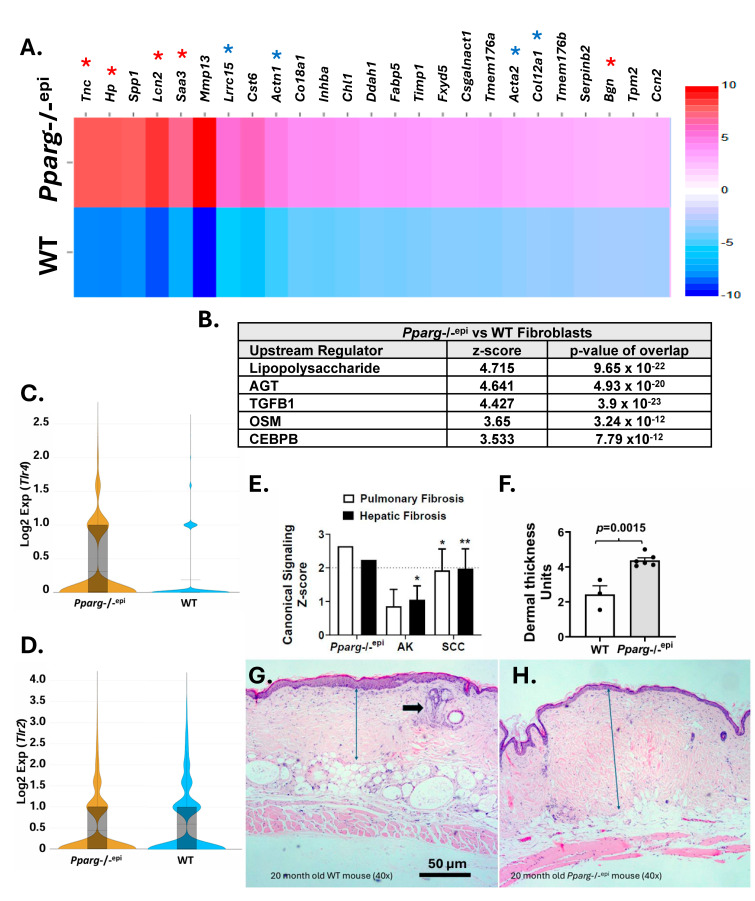
**Single-cell data suggest that LPS signaling is a key mediator of the fibroblast phenotype in *Pparg*-/-^epi^ mice.** (**A**–**D**) Dermal fibroblast populations from WT and *Pparg*-/-^epi^ mice were reclustered, then differentially expressed genes were obtained. (**A**) Heat map showing the top 25 transcripts that are upregulated in *Pparg*-/-^epi^ mouse fibroblasts relative to WT mouse fibroblasts. DAMPs associated with TLR4 activation are noted by red asterisks. Gene transcripts associated with cancer-associated fibroblasts are marked with blue asterisks. (**B**) The differentially expressed genes that are associated with *Pparg*-/-^epi^ fibroblasts were uploaded for gene set enrichment analysis of upstream regulators. The top 5 upstream regulators are shown along with their activation z-score and *p*-value of overlap. (**C**,**D**) Violin plot showing the expression of *Tlr4* (**C**) and *Tlr2* (**D**) in *Pparg*-/-^epi^ and WT mouse fibroblasts. (**E**) After uploading DEGs for the whole transcriptome RNA seq dataset for *Pparg*-/-^epi^ mouse skin relative to WT mouse skin [10], as well as the tumor datasets seen in Figure 1A. Following GSEA, activation z-scores for each dataset were obtained for the two canonical signaling pathways associated with fibrosis. Shown are the mean and SEM for the z-score for AKs and SCCs relative to the z-score for *Pparg*-/-^epi^ mouse skin. *, *p* < 0.05; **, *p* < 0.01; 1 sample *t*-test. (**F**) The skin of geriatric WT and *Pparg*-/-^epi^ mice (≥20 months of age) was fixed in formalin and paraffin-embedded. H&E-stained tissue dorsal back skin sections were measured for dermal thickness. Mean and SEM; 2-tailed *t*-test. (**G,H**) Representative 40× photomicrographs of skin from 20-month-old WT (**G**) and *Pparg*-/-^epi^ mouse skin (**H**). The dermal thickness is demonstrated by the line. The presence of sebaceous glands that are only seen in WT mice is shown by the thick arrow in the image (**G**).

Sustained TLR4 activation of fibroblasts promotes increased TGFβ signaling and fibrosis [85,86]. As loss of epidermal *Pparg* activates TLR4 signaling, this suggests a key role for TLR4-TGFβ signaling in the dermal fibroblast phenotype of *Pparg*-/-^epi^ mice. We therefore performed a recluster analysis focusing solely on fibroblast populations from WT and *Pparg*-/-^epi^ mouse skin. Figure 6A shows the top 25 upregulated genes that differentiate dermal fibroblasts from *Pparg*-/-^epi^ mice relative to wildtype mice. These include the following DAMPs with TLR4 activity: *Tnc*, *Hp*, *Saa3*, *Bgn* and the indirect TLR4 activator *Lcn2* (highlighted by red asterisks). The products of these genes are associated with the acute phase inflammatory reaction (*Saa3* and *Hp*), as well as fibrosis (*Lcn2* [87,88] and *Tnc* [89]). For TNC, studies suggest an important role in driving persistent fibrosis in the dermis and other organs through TLR4-dependent fibroblast activation [89].

Gene set enrichment analysis identified LPS and TGFβ1 signaling as top activated upstream regulators in *Pparg*-/-^epi^ fibroblasts (by z-score) (Figure 6B). In Figure 6C, we also show that *Tlr4* expression is increased in *Pparg*-/-^epi^ FBs. In contrast, *Tlr2* expression is roughly equivalent between fibroblasts of WT and *Pparg*-/-^epi^ mice (Figure 6D). This suggests that the increase in predicted LPS signaling in *Pparg*-/-^epi^ FBs is due to both an increase in TLR4 ligand production and TLR4 receptor expression.

Figure 6B shows additional upstream regulators that are predicted to be activated in *Pparg*-/-^epi^ FBs. These include the known profibrotic mediators angiotensin (*Agt*) [90] and oncostatin M (*Osm*) [91]. The transcription factor CCAAT/enhancer-binding protein beta (CEBPB), which has been identified as a common wound fibroblast signature transcription factor [92], was also predicted to be activated in *Pparg*-/-^epi^ fibroblasts. CEBPB activation is also known to promote fibrosis [93].

Since chronic inflammation and fibrosis are features of malignancy, we examined gene set enrichment analysis results for both *Pparg*-/-^epi^ fibroblasts and human AKs and SCCs. In Figure 6E, mean z-scores for the pulmonary fibrosis and hepatic fibrosis canonical signaling pathways exceed or approach the predicted activation z-score cutoff of 2.0 for SCCs. Likewise, these two canonical signaling pathways are predicted to be activated in *Pparg*-/-^epi^ fibroblasts (Figure 6E). These data suggest that fibrosis is a feature of both *Pparg*-/-^epi^ dermis and SCC tumor stroma. We therefore examined the skin of young *Pparg*-/-^epi^ and WT mice and found no evidence of dermal fibrosis or dermal thickening (not shown). However, when we examined the skin of aged mice (>20 months of age), we found a significant increase in dermal fibrosis, as measured by increased dermal thickness (Figure 6F–H). It should be noted that this analysis was performed in *Pparg*-/-^epi^ mice in the outbred SKH1 genetic background. This was done as *Pparg*-/-^epi^ mice in the C57BL6/J background invariably develop severe scratching behavior due to spontaneous inflammatory lesions that require euthanasia for humane reasons at a relatively young age. Mice in the SKH1 background are more resistant to this scratching behavior, and thus we were able to obtain geriatric mice for this analysis. While the data indicate an increase in dermal thickness in *Pparg*-/-^epi^ mice, this change was observed in a relatively low number of geriatric animals, and there is the confounding variable of the change in the genetic background.

**Figure 7:**
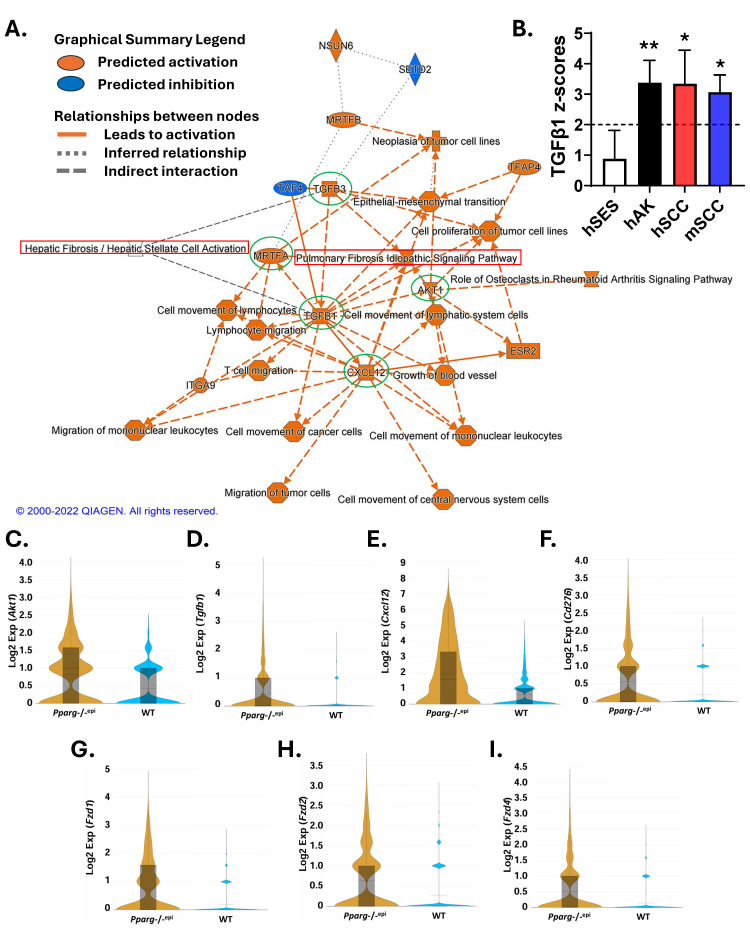
**Transcriptomic analysis predicts that *Pparg*-/-^epi^ fibroblasts will promote fibrosis through TGFβ, MRTFA and CXCL12 signaling.** DEGs from reclustered *Pparg*-/-^epi^ and WT fibroblasts were analyzed by Qiagen Ingenuity Pathway Analysis. (**A**) Graphical summary showing the critical nodal influences of TGFβ1, TGFβ2, AKT1, CXCL12 and MRTFA (green ovals). All nodes positively impact canonical fibrosis signaling pathways (red boxes). (**B**) Mean z-scores for TGFβ1 as an upstream regulator for sun-exposed skin (hSES) relative to non-exposed skin, human AKs (hAKs), human SCCs (hSCCs) and mouse SCCs (mSCCs). Different from 0: *, *p* < 0.05; **, *p* < 0.01; 1-sample *t*-test. (**C**–**I**) Expression data for the specific genes in WT and *Pparg*-/-^epi^ fibroblasts. (**C**) *Akt1*; (**D**) *Tgfb1*; (**E**) *Cxcl12*; (**F**) *Cd276*; (**G**) *Fzd1*; (**H**) *Fzd2*; and (**I**) *Fzd4*.

Figure 7A shows a graphical summary of a gene set enrichment analysis of the differentially expressed genes in *Pparg*-/-^epi^ fibroblasts relative to their WT counterparts. Central regulatory nodes include transforming growth factor (TGF)β1, TGFβ3, protein kinase B isoform 1 (AKT1), C-X-C motif chemokine 12/stromal cell-derived factor-1 (CXCL12), and myocardin-related transcription factor A (*Mrtfa*), all of which are predicted to activate the pulmonary fibrosis idiopathic canonical pathway.

TGFβ1 signaling is well known to promote both fibrotic reactions [94] and myofibroblast differentiation [79]. As increased fibrosis is a feature of many cancers, gene set enrichment analysis of human and mouse tumor datasets shows that the mean activation z-scores for TGFβ1 are elevated only in human and mouse skin cancer, not in sun-exposed human skin (Figure 7B).

MRTFA is necessary to induce smooth muscle actin α2 (*Acta2*) expression during TGFβ-induced myofibroblast differentiation [95]. CXCL12 has also been shown to be a key mediator of fibrosis [96,97]. The predicted activation of AKT1, TGFB1 and CXCL12 signaling are also accompanied by increased expression of the transcripts for *Akt1*, *Tgfb1* and *Cxcl12* in *Pparg*-/-^epi^ fibroblasts relative to WT littermates (Figure 7C–E, respectively).

Given that *Pparg*-/-^epi^ mice are immunosuppressed, it is notable that CXCL12 production by cancer-associated fibroblasts has been shown to mediate resistance to immune checkpoint inhibitor therapy [98]. It is also interesting that the mRNA for the immune checkpoint inhibitor CD276, also known as B7-H3, is upregulated in *Pparg*-/-^epi^ fibroblasts (Figure 7F). Moreover, TGFβ has well-known immunosuppressive activity [99]. Future studies are warranted to determine whether these fibroblasts exhibit immunosuppressive activity.

TGFβ acts through AKT signaling to promote myoFB differentiation [100]. Moreover, TGFβ-induced fibrosis is known to require wingless (WNT)/frizzled (FZD)-dependent activation of β-catenin signaling [101,102]. As β-catenin signaling also triggers myofibroblast differentiation [101], we examined the expression of WNT and FZD isoforms in *Pparg*-/-^epi^ and WT fibroblasts. Fibroblasts from both genotypes expressed similarly high levels of *Wnt2*, with limited expression of other *Wnt* isoforms, except for *Wnt5a*, which was increased in *Pparg*-/-^epi^ fibroblasts (not shown). In addition, FZD isoforms (*Fzd1*, *2* and *4*) all showed increased expression in *Pparg*-/-^epi^ fibroblasts (Figure 7G–I).

### 2.7. Fibroblast Clusters Specific to Pparg-/-^epi^ Mouse Skin Exhibit Gene Expression Profiles That Are Consistent with Profibrotic Myofibroblasts

We previously demonstrated that *Pparg*-/-^epi^ mouse dermis contained a large number of fibroblasts expressing the myofibroblast (myoFB) marker gene S100 calcium-binding protein A4 (*S100A4;* also known as fibroblast specific protein 1) and alpha smooth muscle actin (*Acta2*) [19]. In Figure 2A, these two myoFB clusters are found in clusters 16 and 20 (bounded by the hashed red line). Both clusters 16 and 20 are seen primarily in *Pparg*-/-^epi^ mice, and these clusters are also major sources of many of the genes that differentiate the transcriptome of *Pparg*-/-^epi^ mouse skin (Figure 2B and Figure S2A–K).

**Figure 8:**
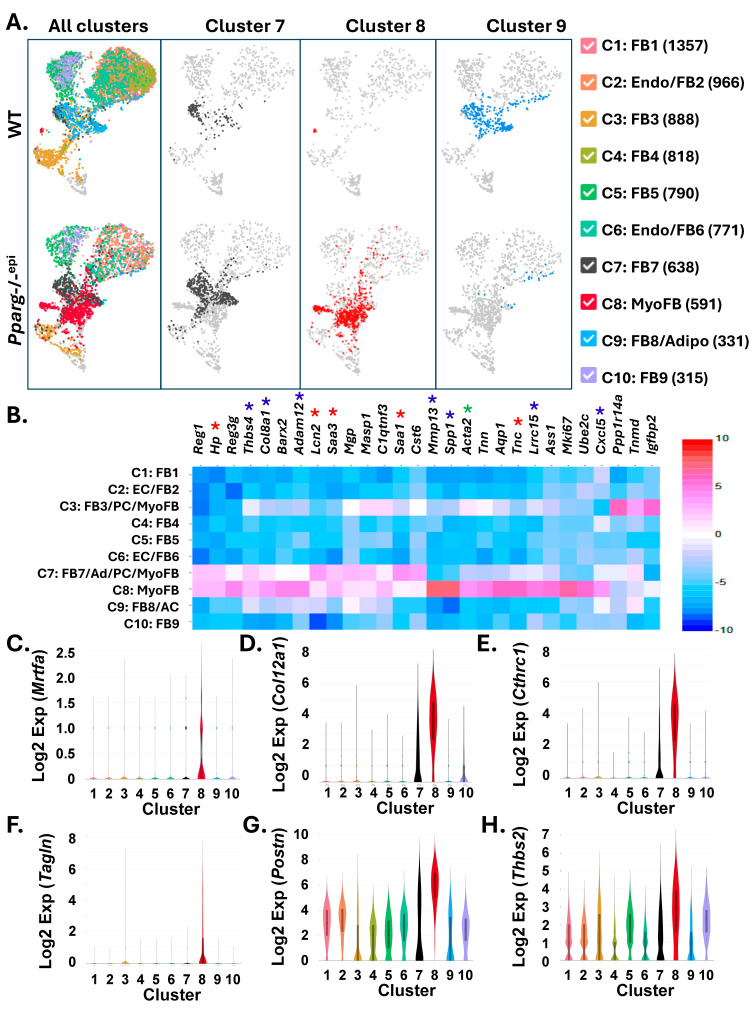
***Pparg*-/-^epi^ mouse skin features two fibroblast populations with inflammatory and myoCAF genetic markers.** (**A**) Cells were selected using the following filters (*Col1a1+*, *Col1a2+*, *Pdpn+*, *Dcn+*, *Ptprc−*, *Pecam−*, *Krt10−*, *Krt14−*, *Nes−*, *Tyrp1−*, and *Cd3e−*) using Loupe Browser, then reclustered. The left panel shows a UMAP plot for the reclustered cells that are separated by genotype (WT vs. *Pparg*-/-^epi^). We then focused on the three clusters (clusters 7–9) that are bounded by the hashed red oval in (**A**) and also represent a reclustering of the original myoFB clusters 16 and 20 that we observed in the similar hashed red oval in Figure 2A. The three panels to the right depict the number of cluster 7, 8 and 9 cells that were identified in WT or *Pparg*-/-^epi^ mouse skin. Abbreviations: Ad—adipocyte; FB—fibroblast; EC—endothelial cell; PC—pericyte; myoFB—myofibroblast. (**B**) Heap map showing top cluster-specific differentiating genes. Genes expressing DAMPs: *Hp*, *Lcn2*, *Saa3*, and *Tnc* (red asterisks). The myofibroblast-specific gene *Acta2* is highlighted by the green asterisk. Genes overexpressed in cancer-associated fibroblasts are highlighted by blue asterisks (*Thbs4*, *Col8a1*, *Adam12*, *Mmp13*, *Spp1*, *Lrrc15*, and *Cxcl5*). (**C**–**H**) Cluster 8 cells are enriched in additional marker genes of myofibroblasts: (**C**) *Mrtfa*, (**D**) *Col12a1*, (**E**) *Cthrc1*, (**F**) *Tagln*, (**G**) *Postn* and (**H**) *Thbs2*.

Given the differences in key fibrosis-related genes in *Pparg*-/-^epi^ fibroblasts, as well as the presence of potential myofibroblasts in these mice, we performed a recluster analysis of all fibroblasts in both WT and *Pparg*-/-^epi^ dermis. Following reclustering, 10 fibroblast clusters were identified (Figure 8A). Enrichr analysis indicated that all 10 clusters represented fibroblasts (Figure S5A). In addition, clusters 2 and 6 also likely contained endothelial cells, clusters 3 and 7 contained some cells expressing pericyte-specific genes, while clusters 7 and 9 also contained cells with adipocyte features (Figure S5A). Features consistent with pancreatic stellate cells were also annotated to clusters 3, 7 and 8. Pancreatic stellate cells represent profibrotic myoFBs that are present in chronic pancreatitis and pancreatic cancer [103].

The recluster analysis also merged clusters 16 and 20 that were bounded by the hashed red oval in Figure 2A into a single cluster 8 in Figure 8A (hashed red oval). The new cluster 7 also appears to represent a *Pparg*-/-^epi^ mouse-specific merger of the original clusters 3 and 10 from Figure 2A. Cluster 9 in Figure 8A also appears to represent a merger of clusters 3 and 10 from Figure 2A and are found primarily in WT mice.

In Figure S5B–D, we examined the cluster-specific expression of three myoFB marker genes: smooth muscle actin α-2 (*Acta2*), actinin alpha 1 (*Actn1*) [104], and S100a4. All three gene markers were highly expressed in cluster 8, with smaller amounts in cluster 7. While Enrichr analysis indicated that cluster 3 contained markers that annotated to pancreatic stellate cell (Figure S5A), known myofibroblast markers *Acta2* and *Actn1* were largely absent in this cluster.

Since myofibroblasts are highly fibrogenic, we further examined clusters 7 and 8. Figure 8A shows that these myofibroblast clusters are found primarily in *Pparg*-/-^epi^ mice. Not surprisingly, these two clusters primarily expressed DAMPs that were found to be differentially expressed in *Pparg*-/-^epi^ mouse dermal fibroblasts (marked by red asterisks in Figure 8B). Cluster 8 cells also expressed high levels of matrix metalloproteinase 13 (*Mmp13*), osteopontin (*Spp1*) and *Cxcl5* (Figure 8B; blue asterisks). In mouse skin tumors, MMP13 expression was identified in bone-marrow-derived ACTA2+ myoCAFs that were associated with tumor invasiveness [105]. Osteopontin is known to promote myofibroblast differentiation [106], while *Cxcl5* is found to be overexpressed in CAFs [107] as well as fibroblasts present in perturbed mouse tissues [108].

In Figure 7A, GSEA predicted that MRTFA is an upstream regulatory control hub in *Pparg*-/-^epi^ fibroblasts. MRTFA is also thought to drive the formation of myofibroblast cancer-associated fibroblasts (myCAFs) [95,109]. In Figure 8C, we show that *Mrtfa* is expressed primarily in cluster 8 myoFBs.

Cluster 8 cells also expressed several additional marker genes for myofibroblasts. These include leucine-rich repeat containing 15 (*Lrrc15*), A disintegrin and metalloproteinase 12 (*Adam12*), collagen type VIII alpha 1 chain (*Col8a1*), and thrombospondin 4 (*Thbs4*) (Figure 8B; blue asterisks). This gene expression signature parallels that observed for a subcluster of *Lrrc15*-expressing mouse myofibroblasts that are found only in mouse tissues with perturbed states such as fibrosis, skin wound healing, arthritis and cancer [108].

Others have shown by single-cell RNAseq that *Lrrc15*-expressing myCAFs are found in various malignancies but are absent or low in normal tissues [77]. As *Lrrc15* is a TGFβ-induced gene, this study also demonstrated that *Lrrc15*+ myCAFs exhibit a TGFβ gene signature and overexpress *Tgfb1* and *Tgfb3* [77]. Additional signature genes for the LRRC15+ myCAFs included *Adam12* (Figure 8B), *Col12a1* (Figure 8D), collagen triple helix repeat containing 1 (*Cthrc1*) (Figure 8E), transgelin (*Tagln*; Figure 8F), periostin (*Postn*; Figure 8G) and thrombospondin 2 (*Thbs2*) (Figure 8H) [77].

**Figure 9:**
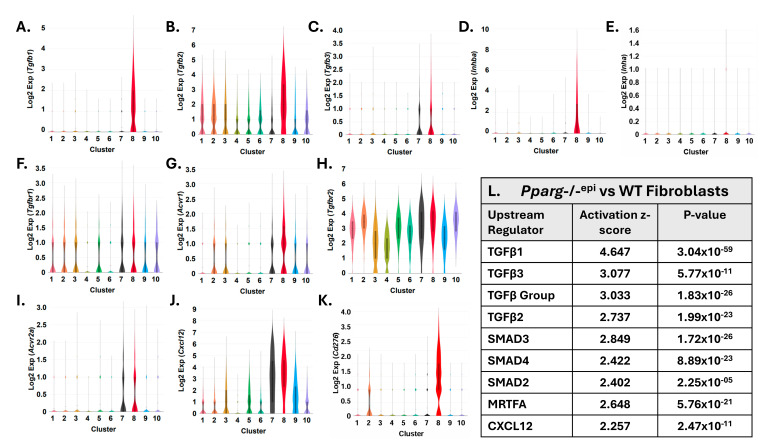
**Cluster-specific expression of profibrotic and immunosuppressive genes in *Pparg*-/-^epi^ dermal fibroblasts.** (**A**–**I**). The 10 cell clusters from the recluster shown in Figure 8 were analyzed for the expression of genes associated with TGFβ-family member signaling. Violin plots are shown demonstrating the expression of *Tgfb1* (**A**), *Tgfb2* (**B**), *Tgfb3* (**C**), *Inhba* (**D**), *Inha* (**E**), *Tgfbr1* (**F**), *Acvr1* (**G**), *Tgfbr2* (**H**), *Acvr2a* (**I**), *Cxcl12* (**J**), and Cd276 (**K**). (**L**) Following the reclustering of fibroblast populations from the scRNAseq dataset, the differentially expressed transcripts from *Pparg*-/-^epi^ relative to wildtype (WT) mouse skin were analyzed by Qiagen Ingenuity Pathway Analysis for upstream regulator influences. The top activated upstream regulators in *Pparg*-/-^epi^ mouse skin are shown with their predicted activation z-score and *p*-value.

As LRRC15+ myoCAFs also exhibit a TGFβ gene signature [77], we examined the expression of TGFβ family members and their receptors. In Figure 9A–E respectively, we show that *Tgfb1*, *Tgfb2*, *Tgfb3*, and activin A (*Inhba*) but not inhibin (*Inha*) transcripts are all enriched in cluster 8. Both activin A and TGFβ1 are thought to play a role in myofibroblast-induced fibrosis [110].

Canonical TGFβ1-3 or activin A signaling results from their binding to type 2 TGFβ receptors (*Tgfbr2*), thereby phosphorylating and activating type 1 TGFβ receptors (*Tgfbr1* (ALK5), *Acvr1b* (ALK4), *Acvr1* (ALK1) or *Acvr1a* (ALK2)) (reviewed in [111]). In Figure 9F,G, type 1 receptors *Tgfbr1* (ALK5) and *Acvr1* (ALK1) are shown to be expressed in clusters 7 and 8 as well as other FB clusters. Activin A preferentially binds to ALK4 but can also serve as a ligand for ALK5 [112]. However, ALK4 (*Acvr1b*) was minimally expressed in all fibroblast clusters (data not shown). Clusters 7 and 8 also expressed type 2 receptors *Tgfbr2* and *Acvr2a* (Figure 9H,I), of which *Acvr2a* was found to be expressed predominantly in clusters 7 and 8.

Using GSEA of transcriptomic data from *Pparg*-/-^epi^ fibroblasts, CXCL12 is predicted to be a central regulatory hub that acts downstream of TGFβ1 and is also predicted to positively impact the pulmonary fibrosis idiopathic signaling (Figure 7A). Cxcl12 is also highly enriched in *Pparg*-/-^epi^ fibroblasts relative to WT fibroblasts (Figure 7E). We therefore examined which fibroblast subclusters expressed *Cxcl12*. In Figure 9J, we show that *Cxcl12* expression is highly enriched in the cluster 7 and 8 fibroblasts that exhibit myofibroblast features and that are largely limited to *Pparg*-/-^epi^ mice. This is of interest as CXCL12 exhibits profibrotic activity [96] and is expressed in CAFs [98].

In Figure 7F, we showed that the immune checkpoint inhibitor B7-H3 (CD276) is overexpressed in *Pparg*-/-^epi^ fibroblasts. In Figure 9K, this is shown to be due to its predominant expression in cluster 8 fibroblasts. In gastric cancer, B7-H3 expression is shown to be increased in both cancer cells and ACTA2-expressing CAFs and is associated with a poor prognosis [113]. siRNA-induced knockdown of B7-H3 in isolated CAFs resulted in a significant reduction in various cytokines, including CXCL12 [113]. Similarly, in renal cancer CAFs, B7-H3 promotes CXCL12 expression, suppresses the apoptosis of CAFs and promotes the invasion and metastasis of renal cancer cells [114].

Interestingly, TGFβ1 has been shown to upregulate the expression of the immune checkpoint inhibitor B7-H3 (CD276) in colorectal cancer cells [115]. Thus, future studies are warranted to examine whether TGFβ signaling drives a B7-H3/CXCL12 signaling pathway in *Pparg*-/-^epi^ dermal fibroblasts that leads to dermal fibrosis and possibly immune suppression.

The above data indicate that TGFβ1-3, activin A and their respective receptors are prominently expressed in *Pparg*-/-^epi^ mouse fibroblasts. We therefore examined the IPA Upstream Regulators that are predicted to be activated (z-score > 2) in *Pparg*-/-^epi^ fibroblasts. In Figure 9L, we show that TGFβ1-3 and TGFβ group signaling are all predicted to be activated (z-scores of 2.373–4.647). In canonical TGFβ signaling, TGFβ1-3 or activin A act to alter gene expression through Smads 2-4 [111]. We also found that Smad2-4 signaling was also predicted to be activated, with z-scores ranging from 2.402 to 2.849. Likewise, consistent with the graphical summary of the *Pparg*-/-^epi^ GSEA (Figure 7A), downstream MRTFA and CXCL12 signaling are also predicted to be activated, with a z-score of 2.648 and 2.257, respectively. Thus, these data suggest that a significant subset of dermal fibroblasts in *Pparg*-/-^epi^ mice are myoFBs that are typically found in the chronic inflammatory environment that is characteristic of fibrotic diseases, wound healing and cancer.

## 3. Materials and Methods

### 3.1. Animal Studies

The derivation of mice lacking epidermal *Pparg* in the C57BL/6 background (*Pparg*-/-^epi^) and wildtype controls (lacking Cre recombinase) was previously described [9]. Mice were housed under specific pathogen-free conditions at the Indiana University School of Medicine. The study was conducted according to the guidelines of the Declaration of Helsinki and approved by the Institutional Review Board (Institutional Animal Care and Use Committee) of the Indiana University School of Medicine and the Richard L. Roudebush VA Medical Center (protocol IN-1128, approved on 9 August 2019, and IN-23005, approved on 21 March 2023).

### 3.2. Whole Transcriptomic RNA Sequencing

RNA sequencing and subsequent differentially expressed gene analysis was performed on epidermal scrapings of six *Pparg*-/-^epi^ mice relative to six wildtype controls as previously described [10]. Briefly, scraping was performed in skin frozen under dry ice until pink-tinged tissue was observed to ensure that the epidermis was removed up to the subepidermal microvascular bed. Total RNA was extracted, and RNA integrity numbers (RIN) were assessed. No significant difference was observed in the RIN numbers between the two genotypes. Library construction was performed using 200 ng of total RNA with a KAPA mRNA HyperPrep Kit (KK8581, ΔKapa Biosystems, Roche Sequencing and Life Science, Wilmington, MA, USA). Paired-end sequencing (2 × 75 bp) was performed by the Center for Medical Genomics at the Indiana University School of Medicine using an Illumina HiSeq 4000 (Illumina Inc., San Diego, CA, USA). The number of reads that fell into different annotated regions (exonic, intronic, splicing junction, intergenic, promoter, UTR, etc.) of the reference genes were determined, and low-quality mapped reads (including reads mapped to multiple positions) were excluded. Sequencing alignment was performed by mapping to the mouse reference genome (mm10/refGene). Differential gene expression analysis was performed with edgeR (version 3.22.5) [88]. In this workflow, the statistical methodology applied uses negative binomial generalized linear models with likelihood ratio tests. This dataset is publicly available at the Gene Expression Omnibus (GEO), National Center for Biotechnology Information (NCBI) depository (Accession number: GSE164024).

### 3.3. Single-Cell Isolation, Sequencing, Library Prep and Analysis

Cells were isolated from the normal dorsal back skin of one male and one female for each of the *Pparg*-/-^epi^ and wildtype genotypes in the C57BL6/J background as previously described [19]. Single-cell sequencing, including cell barcode and UMI sequences, and 100 bp RNA reads were generated with Illumina NovaSeq 6000 (Illumina Inc., San Diego, CA, USA) at the Center for Molecular Genomics of the Indiana University School of Medicine as previously described [19]. As previously described [19], raw sequence processing into FASTQ files using CellRanger 6.1.1, alignment with the reference genome mm10 with the RNAseq aligner STAR, and gene expression of each UMI (unique molecular indices) traced to each cell was performed. The filtered feature cell barcode matrices were generated by CellRanger, and ambient RNA was removed using the R package SoupX version 1.5.2 [116]. For initial cell clustering, cell cluster marker gene identification was performed using the R package Seurat version 4.0 [117,118,119,120]. QC metrics for library size, number of features/genes, and mitochondrial reads (based on median absolute deviation (MAD), with 3 MAD used here) were calculated with Scater [121] and combined with the QC analysis in Seurat to determine the parameters used for excluding low-quality cells. Low-quality cells were excluded with the following criteria: cells with unique feature/gene counts over 7000 or less than 300, or >10% reads mapped to mitochondrial genomes. As a quality control check, we compared the DEGs that were significantly altered in *Pparg*-/-^epi^ skin relative to wildtype skin for both the scRNAseq and the whole transcriptomic RNAseq. This comparison revealed 94.94% agreement between the DEGs identified by scNAseq compared with the DEGs obtained by whole transcriptomic RNA-seq [19]. A summary of the final scRNAseq data output has been previously published [19] and has been deposited to the GEO database as accession number GSE320138.

### 3.4. Unsupervised Cell Cluster Segregation and Cluster Identification

Unsupervised clustering was performed using the Loupe Browser software (v6.5.0; 10x Genomics, Pleasanton, CA, USA) by uniform manifold approximation and projection (UMAP). Using Loupe Browser, DEGs for each individual cell cluster were obtained. For cluster cell-type annotation, each cluster was differentiated from all other cell clusters in the dataset using the “Globally Distinguishing” gene feature comparison tool. The cell type within each cluster was identified by uploading the top differentially expressed genes (*p*-value < 0.05) for each cluster into the Enrichr online analysis application [122,123,124], then cross-referencing with the PanglaoDB database [125].

### 3.5. Differentially Expressed Gene Analysis of WT and Pparg-/-^epi^ Cells

For a comparison of the differentially expressed genes for *Pparg*-/-^epi^ relative to wildtype cells of the same cell type, we utilized Loupe Browser to first recluster the cells of interest (e.g., macrophage and dendritic cell clusters). Following reclustering, the differentiating features for the cells of interest were identified using the “Locally Distinguishing” gene feature comparison tool with the category set to condition (genotype). For gene expression features for each subcluster within a reclustered cell type (e.g., heat maps for top differentiating genes per cluster or violin plots for individual gene expression), locally distinguishing features were again analyzed with the category set to include all cell subclusters.

### 3.6. Gene Set Enrichment Analysis

Ingenuity Pathway Analysis (IPA) (Qiagen Inc., Germantown, MD, USA) was performed to identify predicted diseases and biofunctions, canonical pathways and upstream regulators that are enriched in datasets relative to what would be expected by chance (Qiagen Inc., Germantown, MD, USA, https://digitalinsights.qiagen.com/IPA) [126]. IPA allows for individual dataset or multiple dataset comparison analysis. Datasets were uploaded using gene expression analysis cutoffs of expressed Log2 ratios of >Abs (0.6) and a *p*-value or adjusted *p*-value (if available) of <0.05. Individual gene expression was obtained using the expression analysis function of the IPA software. In addition to the mouse skin RNAseq and scRNAseq datasets, a number of tumor datasets were uploaded for analysis. Thus, the IPA URL was accessed multiple times over a time period encompassing August 28. 2022 through June 26, 2025. Heat maps were downloaded as image files, and data files (e.g., activity z-scores, expressed *p*-value, and *p*-value of overlap) were obtained by downloading in Excel data format.

### 3.7. Dermal Thickness

Dermal thickness was measured in aged (>20 months) *Pparg*-/-^epi^ mice in the SKH1 outbred background [8]. This was done as *Pparg*-/-^epi^ mice in the C57BL6/J background invariably develop spontaneous inflammatory lesions, with 75% of *Pparg*-/-^epi^ mice in the C57BL6/J background exhibiting wound lesions from scratching behavior by the age of 18–23 weeks [10]. *Pparg*-/-^epi^ mice in the SKH1 background were used as they did not exhibit the scratching behavior, avoiding variances due to healed wounds. Moreover, obtaining geriatric *Pparg*-/-^epi^ mice in the C7BL6/J background was not possible, as the self-wounding generally resulted in early euthanasia, typically within 5–8 months of age. This change in the background genotype, while necessary due to the constraints noted for the C57BL6/J background, also introduces potential confounding genetic background influences on the analysis. The dermal layer was measured in 3 WT and 6 *Pparg*-/-^epi^ mice. Following euthanasia, the dorsal skin of the mid-back overlying the spine was excised, then formalin-fixed and paraffin-embedded with an on-edge orientation to allow for sectioning perpendicular to the epidermal–dermal plane. After hematoxylin and eosin staining, the distance from the epidermal–dermal junction to the superficial edge of the panniculus adiposus that forms the dermal–hypodermal junction was measured at 10 random fields for each section.

### 3.8. Datasets

The following publicly available transcriptomic datasets from sun-exposed skin, human AKs, human SCCs, or mouse SCCs were analyzed in this study: E-MTAB-5678 [127], GSE98774 (not published), GSE32628 [128], GSE84293 [129], GSE108008 [130], GSE2503 [131], Bailey et al., 2023 [132], GSE45164 [133], GSE125285 [134], Hu et al., 2022 [135], GSE139505 [136], GSE42677 [20], GSE142108 [137], Kita et al., 2016 [138], Zou et al., 2021 [139], GSE11990 [140], GSE19616 [140], GSE63967 [141], GSE84292 [129], and GSE89516 [142]. Additional details regarding these datasets can be found in our previous study [19]. In addition, the following publicly available datasets examined LPS-induced gene changes in macrophages: GSE48609 [143], GSE42190 [144], GSE178478 [145], GSE40029 (not published), GSE192517 [146], and GSE197837 (not published). For datasets with the GSE label, details can also be obtained from the National Center for Biotechnology Information (NCBI) Gene Expression Omnibus (GEO, www.ncbi.nlm.nih.gov/geo/) (accessed on 9 April 2026).

## 4. Conclusions

While cancer develops within an inflammatory microenvironment [12,13], it is unclear how tumor cells orchestrate the inflammatory, immune suppressive and profibrotic changes that are characteristic of the tumor microenvironment. Previously, we showed that mice lacking epidermal *Pparg* are highly susceptible to cutaneous carcinogenesis [8,147,148] and exhibit both increased dermal inflammation and immune suppression [9,10,11]. PPAR signaling is also the top inhibited canonical signaling pathway in human actinic keratoses, as well as human and mouse SCCs, but not human sun-exposed (SE) skin [19]. In human and mouse tumors, this decrease in PPAR signaling was accompanied by a significant reduction in PPARγ mRNA expression [19]. In contrast, in human SE skin relative to non-exposed skin, *PPARG* expression was increased by 50.18% [19]. Similarly, gene set enrichment analysis showed a reduction in PPARγ activity scores in tumors but not sun-exposed skin [19].

We now show that the loss of epidermal *Pparg* in mouse skin is linked to increased pro-inflammatory, immunosuppressive and profibrotic signaling pathways. Our findings in *Pparg*-/-^epi^ skin are consistent with transcriptomic changes seen in actinic disease and cutaneous SCCs. This suggests that loss of PPARγ signaling in epidermal tumor cells is key to the tumor–stromal crosstalk that leads to these dermal and immune cell changes that are associated with neoplasia. The idea that loss of *PPARG* occurs within the tumor cell compartment of NMSC is supported by our analysis of published transcriptomic data from microdissected tumor cells alone [19].

In this study, we show that LPS signaling is the top activated upstream regulator in both *Pparg*-/-^epi^ mouse skin and AKs or SCCs. Moreover, single-cell sequencing revealed that all major cell types in *Pparg*-/-^epi^ mouse skin exhibited a predicted increase in LPS signaling. LPS signaling is mediated by TLR4. Transcripts encoding the TLR4 activators S100A8, S100A9, tenascin C, lipocalin 2 and biglycan were all significantly elevated in AKs and SCCs but were not significantly altered or only modestly increased in SES skin.

We also show that the increase in DAMP expression and the predicted activation of LPS signaling is also observed within this microdissected tumor epithelium dataset. TLR4 activation leads to canonical activation of NF-κB that in turn triggers the transcription of numerous cytokines and chemokines, including DAMPs, that then act to further activate TLR4, both in the cell of origin or other cells [15,16].

This shift to an increase in LPS signaling with loss of PPARγ activity may be a critical component of NMSC formation. TLR4 has drawn interest as a potential target for skin cancer prevention and treatment [16]. In mice, TLR4 expression is directly correlated with UV-induced tumor yield, inflammatory mediator expression and the recruitment of CD11b^+^Gr-1^+^ myeloid cells [17]. Moreover, mice lacking *Tlr4* or *Myd88* are resistant to chemical skin carcinogenesis [149]. In this model, endogenous TLR4 ligands are likely necessary, as carcinogen-induced inflammation was not dependent on increased bacterial LPS [149]. Other groups have shown that topical administration of small molecule TLR4 antagonists were an effective preventive agent for photocarcinogenesis in mice [150,151]. Thus, TLR4 antagonists are currently in clinical trials for melanoma, NMSC and Merkel cell carcinoma [reviewed in [16]].

While TLR4 antagonists are in trials for their potential anti-cancer effect, it should be noted that mice lacking TLR4 show a reduced immune response and increased mortality in response to Salmonella infection [152]. However, long-term photocarcinogenesis studies in mice [150,151] and short-term studies of humans with severe sepsis [153] have failed to reveal safety concerns for TLR4 inhibitors.

TLR4, TNF and TLR3 are all produced in response to NF-κB signaling and in turn activate NF-κB [154]. These cytokines were predicted to be the top 3 activated upstream regulators in *Pparg*-/-^epi^ mice as well as tumors but not SE skin. Thus, unrestrained production of these cytokines could play a role in a feed-forward signal that maintains a chronic inflammatory state following the loss of tumor or epidermal PPARγ activity.

A weakness of our studies is that our mouse model results in embryonic loss of *Pparg*. Thus, our studies done on adult mice would suffer from long-standing dermal inflammatory changes that would create a new normal state. Thus, the proximal events that are initiated by loss of epidermal PPARγ cannot be assessed. If loss of *PPARG* is a defining feature of NMSC tumor–stromal interactions, then it would be important to determine the nature of these early signals to identify potential interventional targets.

Another finding from our single-cell sequencing data is that loss of epidermal *Pparg* in mice results in an accumulation of neutrophils and macrophage cells that express high levels of DAMPs, particularly the calgranulins. S100A8/S100A9 have been linked to an immunosuppressive tumor microenvironment and MDSC chemotaxis [155,156,157]. In studies of UV-induced photocarcinogenesis, the TLR4 antagonist TAK-242 blocked the accumulation of myeloid cells expressing MDSC markers as well as T-cells expressing regulatory T-cell markers [151]. This supports the idea that loss of PPARγ in normal or neoplastic keratinocytes could trigger immune suppression and photocarcinogenesis through a TLR4-dependent accumulation of immunosuppressive MDSCs. This idea may not be limited to skin as mice expressing dominant negative *Pparg* (dn*Pparg*) in type II pulmonary alveolar cells stimulate the mobilization and recruitment of myeloid cells with MDSC activity [158].

We show that macrophages and dendritic cells in *Pparg*-/-^epi^ mice have transcriptomic profiles consistent with the loss of PPAR signaling. Moreover, mice expressing the dn*Pparg* transgene in myeloid cells also results in the systemic accumulation of immunosuppressive MDSCs, increased inflammation and tumorigenesis [159]. Since *Pparg*-/-^epi^ mice lack *Pparg* only within keratinocytes, the loss of PPAR signaling in stromal inflammatory cells must occur through indirect paracrine signaling. Studies in adipocytes, mesenchymal stem cells and macrophages show that stimulation with LPS or TNFα act to suppress *PPARG*/*Pparg* transcript expression [160]. Thus, downregulation of PPARγ activity by sustained TLR4 ligand and TNFα-dependent NF-κB signaling could result in loss of myeloid cell PPARγ activity and subsequent MDSC polarization. Additional studies are needed to determine whether the macrophage populations found in *Pparg*-/-^epi^ mice play a role in the immune suppression and increased susceptibility to chemical and photocarcinogenesis that are observed in these mice.

PPARγ agonists have been demonstrated to have strong anti-fibrotic activity in chronic inflammatory diseases such as systemic sclerosis [94,161]. Our single-cell sequencing demonstrated that the loss of epidermal *Pparg* results in significant changes to dermal fibroblasts. Multiple profibrotic DAMPs were highly expressed in these fibroblast populations. Moreover, chronic TLR4 stimulation in skin induces not only inflammation but also TGFβ-dependent gene expression and fibrosis [34]. Notably, several clusters of *Pparg*-/-^epi^ mouse fibroblasts are present that express increased levels of multiple TGFβ family members, profibrotic genes and markers of myofibroblast differentiation. Interestingly, TGFβ has been shown to suppress PPARγ signaling in myofibroblasts [94], raising the possibility that TLR4 and TGFβ activity may act in concert to suppress anti-fibrotic PPARγ signaling in myofibroblasts.

*Pparg*-/-^epi^ mice exhibit a profound loss of contact hypersensitivity responses [9]. In addition, the PPARγ agonist rosiglitazone restores normal CHS responses following UV treatment [9]. As TLR4 activation is known to be necessary for the ability of UV treatment to suppress contact hypersensitivity (CHS) [16], this raises the possibility that PPARγ promotes normal immune responses by regulating downstream TLR4 signaling. In addition, a potential role for TGFβ1 signaling in this loss of cutaneous immune responses is an area of active interest as TGFβ1 signaling acts to suppress anti-tumor immune responses [24]. As PPARγ activation acts to suppress both TLR4 and TGFβ expression and activity [162,163], additional work is necessary to determine whether TLR4 and TGFβ signaling are necessary for the defect in CHS responses seen in *Pparg*-/-^epi^ mice. Future work is also needed to determine whether loss of PPARγ activity in NMSC tumor cells cause increased TLR4 and TGFβ signaling leading to reduced anti-tumor immunity.

In conclusion, our data suggest that loss of PPARγ expression in cancer epithelium would trigger a self-sustaining TLR4- and TGFβ-dependent profibrotic inflammatory and immunosuppressive stromal microenvironment that would provide a rich environment for tumor development.

## Data Availability

The raw data supporting the conclusions of this article were deposited with the National Center for Biotechnology Information Gene Expression Omnibus (GEO). The accession number for our whole transcriptomic mRNA sequencing dataset is GSE164024, while the accession number for our single-cell RNA sequencing dataset is GSE320138.

## Supplementary Materials

The following supporting information can be downloaded at: https://www.mdpi.com/article/10.3390/ijms27094136/s1.

## Abbreviations

The following abbreviations are used in this manuscript:

αSMA/ACTA2	Alpha smooth muscle actin
Adam12	A disintegrin and metalloproteinase 12
ALK1/ALK2/ALK4/ALK5	Activin receptor-like kinase isoforms; type 1 TGFβ receptors (Acvr1, Acvr1a, Acvr1b, and Tgfbr1)
CAF	Cancer-associated fibroblast
CHS	Contact hypersensitivity
Cited2	Cbp/p300 interacting transactivator with Glu/Asp-rich carboxy-terminal domain 2
Col8a1	Collagen type VIII alpha 1 chain
Col12a1	Collagen type XII alpha 1 chain
Cthrc1	Collagen triple helix repeat containing 1
Cxcl12	C-X-C motif chemokine ligand 12
Cxcl5	C-X-C motif chemokine ligand 5
DAMP	Damage-associated molecular pattern
dnPparg	Dominant negative Pparg transgene
GEO	Gene Expression Omnibus (NCBI)
H&E	Hematoxylin and eosin staining
Inha	Inhibin alpha subunit
Inhba	Inhibin beta A; forms activin A
IPA	Ingenuity pathway analysis
Irgm1/IRGM	Immunity-related GTPase M
LPS	Lipopolysaccharide
Lrrc15	Leucine-rich repeat containing 15
MDSC	Myeloid-derived suppressor cell
MMP13	Matrix metalloproteinase 13
Mrtfa	Myocardin-related transcription factor A
MyD88	Myeloid differentiation primary response 88
NCBI	National Center for Biotechnology Information
NES	Non-sun-exposed skin
NF-κB	Nuclear factor kappa-light-chain-enhancer of activated B cells
PDGF	Platelet-derived growth factor
Postn	Periostin
Pparg/PPARγ	Peroxisome proliferator-activated receptor gamma
RAR	Retinoic acid receptor
RPKM/UMI	Unique molecular identifiers for RNA-seq
S100A8/S100A9	Calgranulins
scRNA-seq	Single-cell RNA sequencing
SES	Sun-exposed skin
Spp1	Osteopontin
Tagln	Transgelin
Tgfbr1/Tgfbr2	Type 1 and type 2 TGFβ receptors
Thbs2/Thbs4	Thrombospondin 2 and 4
TLR3	Toll-like receptor 3
TLR4	Toll-like receptor 4
TNF/TNFα	Tumor necrosis factor alpha
UMAP	Uniform manifold approximation and projection
WT	Wildtype
