# Supplementary material for: Epidermal PPARγ Signaling as a Suppressor of Toll-like Receptor-Mediated Inflammation and Fibrosis: Relevance to Cutaneous Squamous Cell Carcinoma
Source: Int J Mol Sci. 2026 May 5;27(9):4136. doi: 10.3390/ijms27094136 (PMC13164369; doi:10.3390/ijms27094136)
Supplement: Supplementary file 1 [file ijms-27-04136-s001.zip › ijms-4173714-supplementary.pdf]

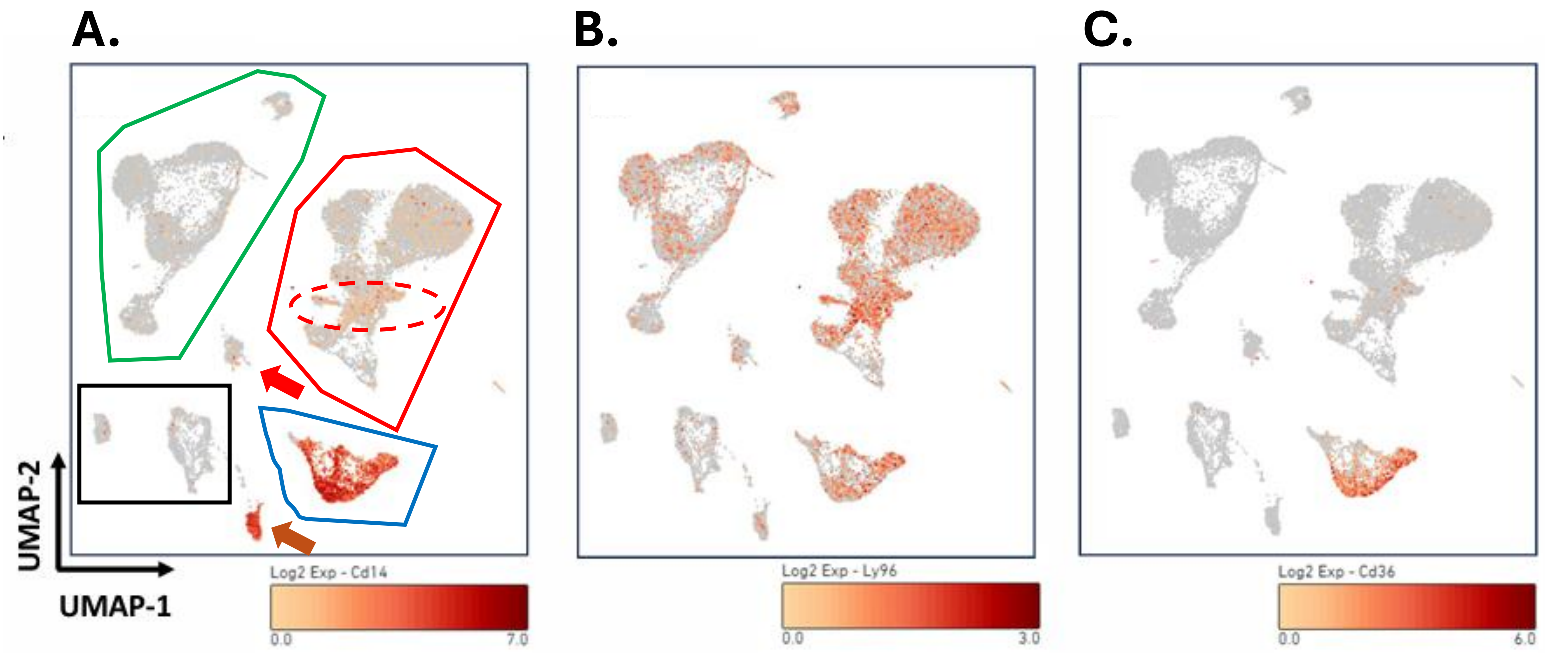

Figure S1: Expression of TLR coreptors in cell clusters of WT and *Pparg*-/-^{epi} mice. UMAP images are shown that demonstrate cluster-specific expression of the following: **A.)** *Cd14*; **B.)** *Ly96* and **C.)** *Cd36*. The color scale key shows the Log2 expressed differences for each transcript. In panel A, colored outlines are used to border specific cell cluster groups: keratinocyte clusters (green); non-immune cell, non-smooth muscle stromal cell clusters (solid red); myofibroblasts (hashed red); non-neutrophil myeloid cells (except for Langerhans' cells) (blue); Cd3+ lymphocytes (black). The brown arrow points to the neutrophil cluster. The red arrow points to the Langerhans cell cluster.

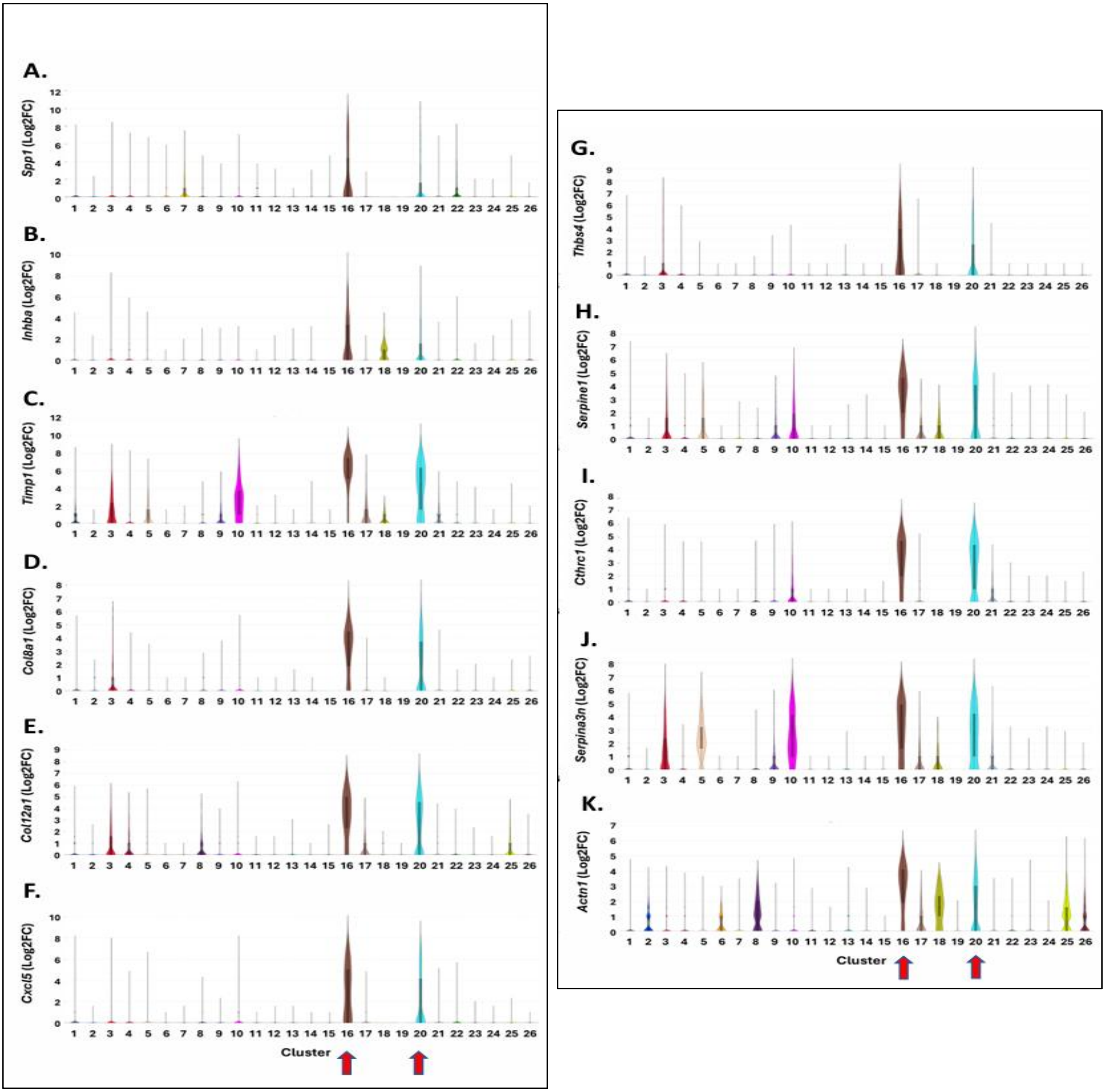

Figure S2: Top differentially upregulated gene transcripts in *Pparg*-/-^{epi} mouse skin are enriched in myofibroblast clusters 16 & 20 (red arrows). Single cell RNA sequencing data from *Pparg*-/-^{epi} mouse skin and wildtype (WT) mouse skin was performed. Top differentially expressed genes in *Pparg*-/-^{epi} mouse skin relative to WT skin were identified. We then performed cluster specific analysis of these key differentially expressed genes using the Gene/Feature Expression function. Data is expressed as the LOG2 expression ratio (Log2FC) and shown as a violin plot. **A.)** *Spp1*; **B.)** *Inhba*; **C.)** *Timp1*; **D.)** *Col8a1*; **E.)** *Col12a1*; **F.)** *Cxcl5*; **G.)** *Thbs4*; **H.)** *Serpine1*; **I.)** *Cthrc1*; **J.)** *Serpina3n*; **K.)** *Actn1*. Myofibroblasts clusters 16 & 20 are futher highlighted by the red arrows at the bottom of panels F and K.

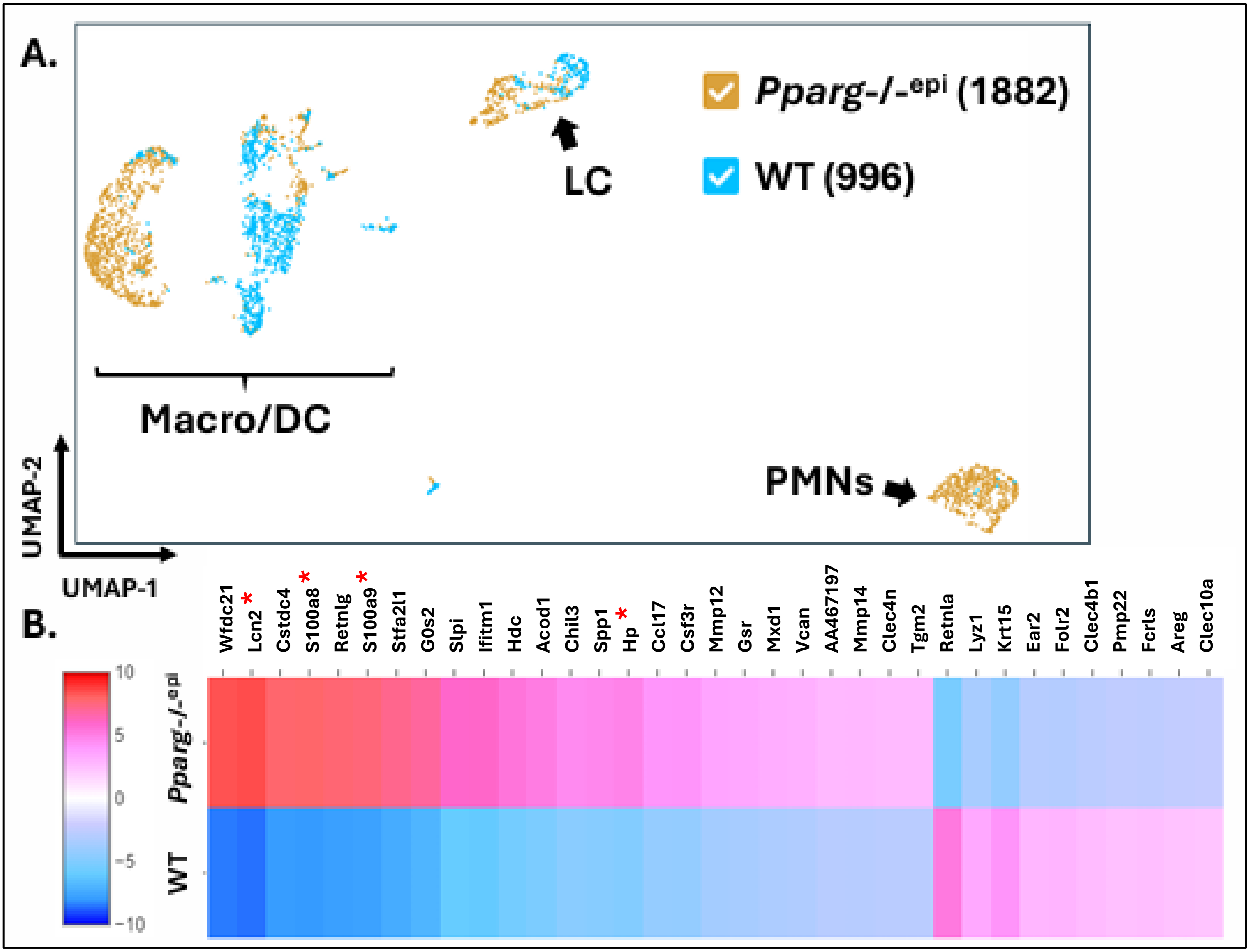

Figure S3: Recluster analysis of myeloid cell populations reveals that WT and *Pparg-/-*^{epi} non-neutrophil myeloid populations are largely segregated and that high DAMP expression is a major feature of *Pparg-/-*^{epi} myeloid cells. Myeloid cell clusters 6, 7, 11, 14 and 22 that are seen in Figure 2A were reclustered. A.) UMAP plot showing the differences in WT (blue) and *Pparg-/-*^{epi} (brown) myeloid cells. Clusters that included macrophages and Langerin- dendritic cells (Macro/DC) showed strong separation of cells clustering to WT or Pparg-/-epi mouse skin. Langerin+ cells (LC) also showed largely separated populations. As there were very few neutrophils (PMNs) isolated from WT mouse skin, an analysis of cluster separation is not possible for neutrophils. **B.)** Heatmap showing the top 25 upregulated genes that differentiate Pparg-/-epi mouse dermal myeloid cells from WT mouse skin. The top 10 genes upregulated in WT mouse skin is also depicted. The color scale depicts gene expression differences as Log2 expressed ratios. DAMPs are highlighted using the red asterisks.

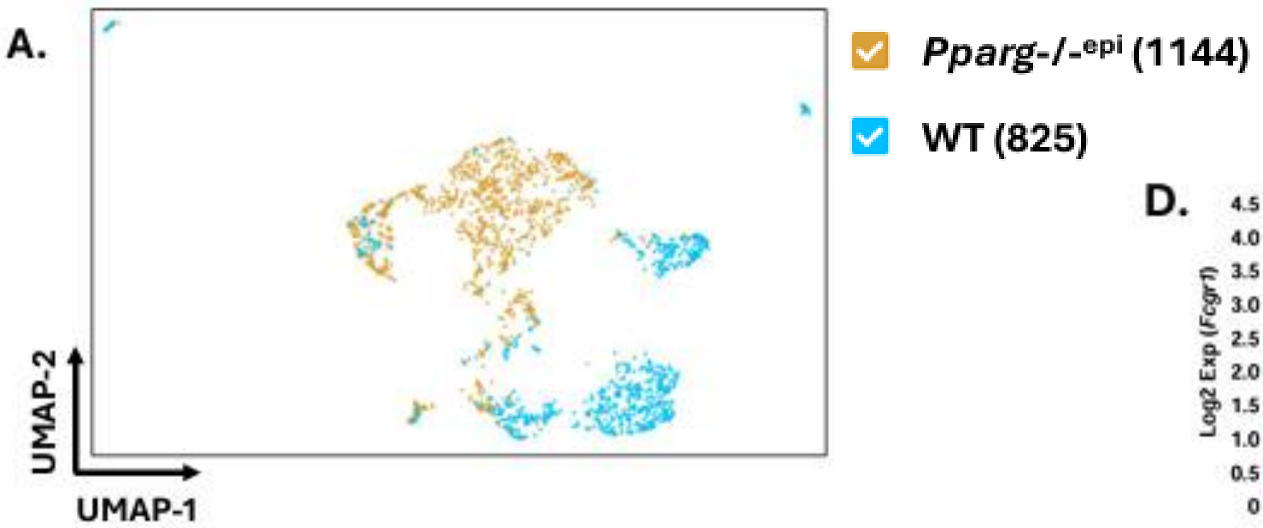

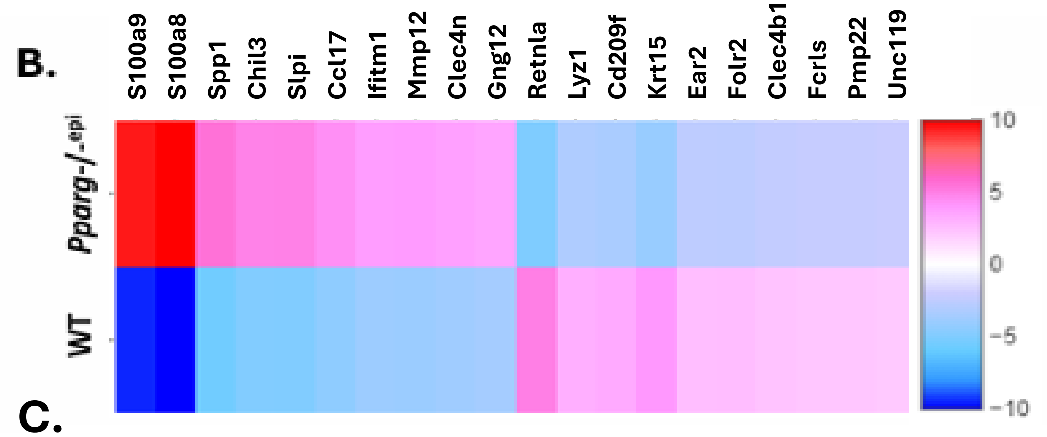

C.

Cluster	Identified Cell Type	Adjusted P-value	Combined Score
1	Macrophages	2.06×10^{-101}	15064.6
	Myeloid-derived suppressor cells	5.53×10^{-58}	7882.8
2	Macrophages	4.27×10^{-86}	10428.4
	Monocytes	1.04×10^{-58}	5164.0
	Dendritic cells	3.85×10^{-61}	5050.6
3	Macrophages	3.41×10^{-78}	10940.5
	Dendritic cells	2.91×10^{-72}	9303.6
	Plasmacytoid dendritic cells	4.29×10^{-56}	6917.8
4	Macrophages	4.00×10^{-81}	12927.8
	Myeloid-derived suppressor cells	9.18×10^{-60}	11599.6
5	Macrophages	1.32×10^{-78}	11218.7
	Microglia	1.41×10^{-56}	6851.5
	Kupffer cells	1.22×10^{-51}	6373.6
6	Macrophages	2.32×10^{-95}	17653.3
	Microglia	2.23×10^{-69}	10689.7
	Monocytes	1.90×10^{-71}	10308.1

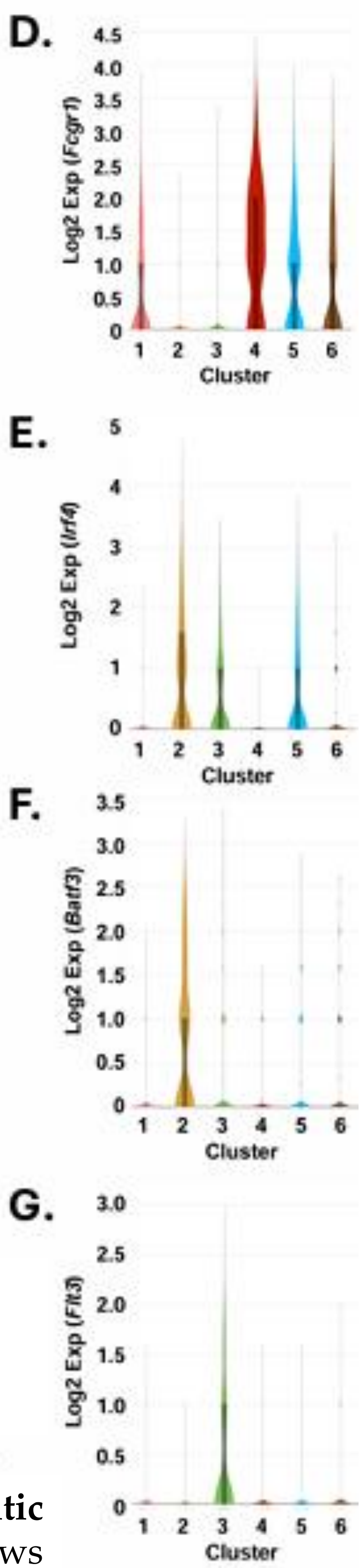

Figure S4: Recluster analysis of macrophage and Langerin- dendritic cells from WT and *Pparg-/-*^epi^ mouse skin. A.) UMAP plot shows largely distinct populations of cells from WT (blue) & *Pparg-/-*^epi^ (brown) mouse dermal macrophages and dendritic cells. **B.)** Heatmap shows that the calgranulins (S100a8 and S100a9) are highly expressed in *Pparg-/-*^epi^ dermal macrophages and dendritic cells. **C.)** Enrichr annotation of the cell types within 6 different myeloid cell subclusters. **D)** Violin plot depicting the cluster-specific expression of the macrophage marker *Fcgr1*. **E-F.)** Violin plots depicting the cluster-specific expression of the dendritic cell markers *Irf4* **(E),** *Batf3* **(F)** and *Flt3* **(G).**

A.

Cluster	Identified Cell Type	Adjusted p-value	Combined Score
1	Fibroblasts	8.63×10^{-09}	304.36
2	Endothelial Cells	5.33×10^{-11}	533.90
	Fibroblasts	5.81×10^{-10}	372.48
3	Fibroblasts	3.90×10^{-23}	1680.58
	Pericytes	1.73×10^{-18}	1398.56
	Pancreatic Stellate Cells	4.88×10^{-18}	1702.22
4	Fibroblasts	3.09×10^{-05}	555.80
5	Fibroblasts	9.77×10^{-19}	1147.18
6	Endothelial Cells	5.82×10^{-03}	129.24
	Fibroblasts	7.37×10^{-03}	90.97
7	Fibroblasts	1.14×10^{-27}	2370.29
	Adipocytes	5.85×10^{-23}	1736.98
	Pericytes	1.75×10^{-18}	1398.56
	Pancreatic Stellate Cells	5.12×10^{-18}	1702.22
8	Fibroblasts	2.05×10^{-32}	3246.87
	Pancreatic Stellate Cells	1.16×10^{-29}	4751.80
9	Fibroblasts	2.36×10^{-15}	978.49
	Adipocytes	1.56×10^{-13}	804.95
10	Fibroblasts	1.11×10^{-13}	635.09

B. **C.** **D.**

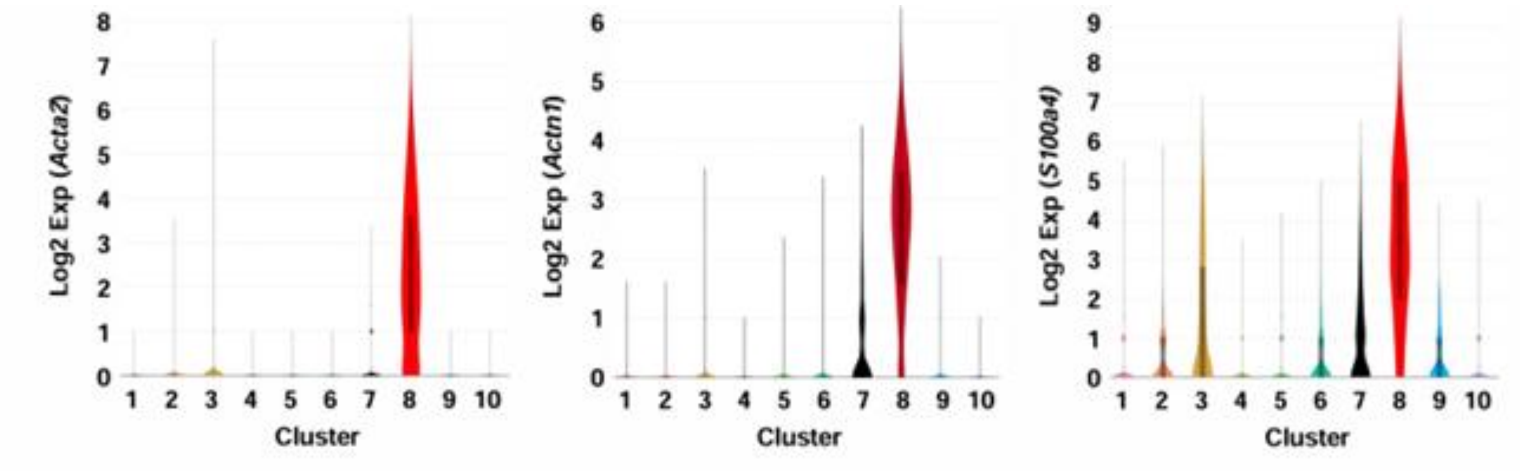

Figure S5: Fibroblast recluster cell identities. Following the recluster of dermal fibroblasts from WT and *Pparg*-/-epi mouse skin, cluster annotation was performed using Enrichr. **A.)** Cell types identified by Enrichr Analysis. **B-D.)** Violin plots demonstrating the Log2 expressed ratio (Log2 Exp) for cluster-specific expression of myofibroblast gene markers *Acta2* **(B)**, *Actn1* **(C)** and *S100a4* **(D)** in *Pparg*-/-epi mouse dermal fibroblasts relative to WT mouse fibroblasts.
